# Analysis of Whole-Genome for *Alternaria* Species Identification

**DOI:** 10.3390/jof11030185

**Published:** 2025-02-26

**Authors:** Ying Yang, Yutong Gan, Wenjie Xu, Yuanhao Huang, Tianyi Xin, Rui Tan, Jingyuan Song

**Affiliations:** 1 School of Life Science and Engineering, Southwest Jiaotong University, Chengdu 610031, China; 2 State Key Laboratory of Bioactive Substance and Function of Natural Medicines, Institute of Medicinal Plant Development, Chinese Academy of Medical Sciences & Peking Union Medical College, Beijing 100193, China; 3 Key Laboratory of Chinese Medicine Resources Conservation, State Administration of Traditional Chinese Medicine of the People’s Republic of China, Engineering Research Center of Chinese Medicine Resource, Ministry of Education, Beijing 100193, China

**Keywords:** Analysis of whole-GEnome, bioinformatics analysis, *Alternaria* species identification, Sanger sequencing, CRISPR-Cas12a

## Abstract

The genus *Alternaria*, functioning as a saprobe, endophyte, and plant pathogen, is widely distributed across various natural and human-impacted environments. Leaf spot and black spot diseases, caused by *Alternaria* species, are the most prevalent plant diseases within this genus, leading to significant reductions in crop yields and substantial economic losses. To facilitate the timely detection of *Alternaria* species during the early stages of infection, enable targeted treatments, and mitigate associated damages, we employed a species identification method based on Analysis of whole-GEnome (AGE). We downloaded 148 genomes, including 31 *Alternaria* species, from the NCBI GenBank database. Through bioinformatics analysis, we constructed a specific-target sequence library and selected a representative sequence per species. The specific target sequences of the seven exemplary *Alternaria* species were subsequently used for validation and rapid detection, utilizing Sanger sequencing and CRISPR-Cas12a technology, respectively. The results demonstrated that our method accurately identified the target species. Additionally, by combining Enzymatic Recombinase Amplification (ERA) with CRISPR-Cas12a, we achieved rapid and precise identification of genomic DNA samples, with a detection limit as low as 0.01 ng/µL within 30 min. Therefore, AGE proves to be a highly robust and efficient method for the detection of *Alternaria* species, offering broad potential for various applications.

## 1. Introduction

The genus *Alternaria* is recorded with 756 species in the Species Fungorum database (https://www.speciesfungorum.org, accessed on 21 December 2024), but due to the unresolved taxonomy of the genus, only 360 species are formally recognized [1,2,3]. This genus is distributed globally, occurring in various natural and anthropogenic environments. It acts as a saprobe, endophyte, and plant pathogen [4,5]. More than 95% of *Alternaria* species are facultative plant pathogens, causing diseases in nearly 400 plant species, primarily leaf spot and black spot diseases. These diseases lead to significant yield losses and economic decline [6,7]. For example, *A. longipes* is a major pathogen responsible for tobacco brown spot disease, one of the most destructive fungal diseases affecting tobacco crops. It is also capable of infecting a wide range of plants, animals, and humans [8]. *A. gaisen* primarily causes black spots on pear fruit surfaces, and pears exported from China to Brazil are required to undergo quarantine for this pathogen [9]. *A. brassicae* primarily infects plants in the Brassicaceae family, including canola, mustard rape, and cauliflower [10]. *A. triticina* causes wheat leaf spot disease and is classified as a high-risk quarantine pest for imported wheat in China, requiring rapid and accurate detection [11]. *A. alternata* is responsible for leaf spots, rotting, and blight in over 380 plant species [12]. These *Alternaria* species have a substantial impact on the yield and quality of agricultural crops. Recent reports have indicated that many plants have been recently infected with *Alternaria*, leading to leaf spot diseases, which have attracted significant attention from the scientific community [13,14,15,16]. *Alternaria* species produce toxic secondary metabolites that pose serious food safety risks to animals and plants [16,17]. The secondary metabolites of *Alternaria* also have medicinal properties, including antibacterial, anticancer, antioxidant, and phytotoxic activities, which are valuable for pharmaceutical, industrial, and medical applications [18,19].

Based on these molecular data, the genus *Alternaria* is classified into 29 sections and seven monotypic lineages [20,21] Significant morphological plasticity within the genus *Alternaria* complicates identification based on morphology alone [22,23]. DNA barcoding technology has become the most widely applied method in the field of molecular identification [24,25]. However, the genomic regions commonly used for species identification show limited differentiation in small-spored species, making them difficult to distinguish [26]. The internal transcribed spacer (ITS), a universal barcode for fungal identification, struggles to distinguish closely related fungi [27]. For example, there is a high degree of similarity in the ITS sequences among *A. brassicae*, *A. alternata*, *A. porri*, *A. infectoria*, and *A. tenuissima*, making it difficult to differentiate them based solely on this region [28]. Even with multi-gene phylogenetic analyses, reliable differentiation of morphologically similar species remains problematic [29]. Related studies have shown that the use of nine commonly used gene regions for species differentiation within the genus *Alternaria* accurately identifies only 11 species, while as many as 35 morphologically similar species cannot be effectively distinguished [29]. Additionally, although secondary metabolites can be used for species identification in some cases, this method also failed to effectively distinguish similar species such as *A. alternata*, *A. arborescens,* and *A. tenuissima* [30,31,32,33]. Some researchers have suggested merging *A. alternata* and *A. tenuissima* into one [29,34,35], while other studies indicated that they can be distinguished through multi-gene sequence analysis [36,37]. Despite their similarities, *A. alternata* and *A. tenuissima* differ significantly in biological characteristics and drug resistance. Therefore, accurately distinguishing these two species and applying targeted treatments was crucial for improving disease management [38]. Another critical issue in *Alternaria* species identification was the incomplete resolution of its taxonomy, with unclear boundaries between species. Some researchers suggested that morphologically and molecularly similar species should be merged into a single species, while other studies indicate that these species can be distinguished through multi-gene analysis [39]. Fortunately, with the rapid advancement of whole genome sequencing technologies, the cost of DNA sequencing has significantly decreased. The sequencing cost per gigabase at the National Human Genome Research Institute (NHGRI) has been reduced from USD 5292.30 in 2001 to just USD 0.006 in 2022 (https://www.genome.gov/). The reduction in sequencing costs will make whole genome analysis an important tool for species identification, significantly improving the accuracy and efficiency of species recognition [40]. For example, the advancement of high-throughput sequencing technologies has been widely applied in plant genomics [41].

**Figure 1:**
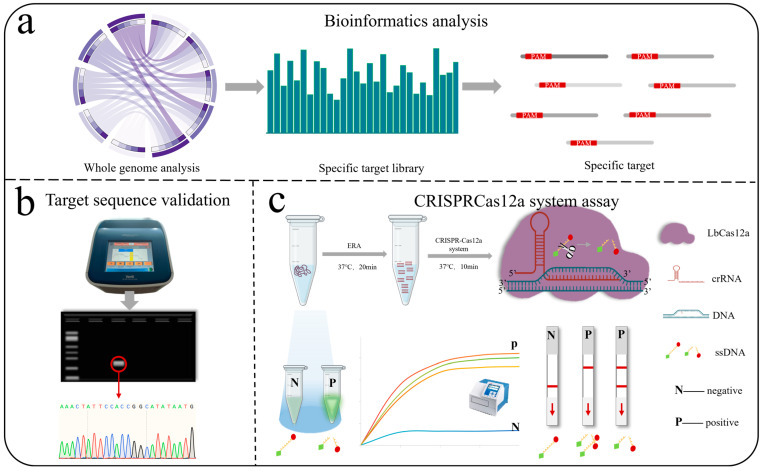
The AGE workflow consists of two main steps: bioinformatics analysis and experimental validation (Sanger sequencing and CRISPR-Cas12a). (**a**) Bioinformatics Analysis: A 25 bp species-specific target sequence library containing protospacer adjacent motifs (PAM) is constructed for each species of *Alternaria*. One sequence is randomly selected and subjected to BLAST alignment in NCBI to identify a reference sequence for experimental validation. Circos analysis was performed using the OmicStudio tools at https://www.omicstudio.cn/tool/, accessed on 31 December 2024. (**b**) Sanger sequencing: Samples are PCR-amplified, then bands from the agarose gel are sequenced and aligned. (**c**) ERA: Efficient and rapid amplification of DNA samples; CRISPR-Cas12a: crRNA is synthesized, followed by a rapid reaction using the one-tube method, and then visual fluorescence detection, fluorescence microplate reader, and lateral flow strips.

In the past two years, a new species identification method, called Analysis of whole-GEnome (AGE), has been proposed. The principle of AGE is that the whole genome sequences of different species inevitably exhibit differences [42]. AGE has been preliminarily applied to several animals, plants, fungi, and their closely related species [43,44,45,46,47]. Figure 1 showed that the workflow of AGE. This method initially used bioinformatics techniques to identify species-specific target sequences, which were then detected in samples using experimental approaches for accurate species identification. In this study, a species-specific target sequence library for *Alternaria* species was constructed, and seven *Alternaria* species—*A. arbusti*, *A. infectoria*, *A. solani*, *A. tenuissima*, *A. triticina*, *A. alternata*, and *A. longipes*—were selected as experimental validation subjects due to their significant economic and ecological importance in agriculture. According to the NCBI taxonomy database, *A. arbusti*, *A. infectoria*, and *A. triticina* belonged to the *Infectoriae* section. *A. tenuissima*, *A. alternata*, and *A. longipes* were classified under the *Alternaria* section. *A. solani* was classified under the *Porri* section. We successfully identified these seven species using Sanger sequencing and CRISPR-Cas12a technology. This study demonstrated that the AGE provides a high degree of accuracy and reliability in species identification, particularly for closely related species with similar morphology, highlighting its significant potential for application in species identification.

## 2. Materials and Methods

### 2.1. Collection of Genome Sequences for the Target Species

Genome data for all existing *Alternaria* species were downloaded from NCBI until 1 May 2024, totaling 165 genomes. The phylogenetic analysis excluded certain genome data embedded within other species, as these genomes lacked sufficient evidence to support their correct species classification and were sourced from institutions not primarily focused on fungal research. Ultimately, 31 species and 145 genomes were included in the analysis. Detailed information on the strains, genome assembly numbers, and submitting institutions was provided in Supplementary Table S1.

### 2.2. Bioinformatics Screening of Specific Target Sequences

#### 2.2.1. Construction of a Species-Specific Target Library for Alternaria

This study utilized an internally developed software tool, Analyzer of whole-GEnome v1.0 (Registration number: 2025SR0250433), which is currently undergoing a software copyright application. The genome sequences of all species were fragmented into 25 bp segments to construct a fragment sequence library for the genus *Alternaria*. From these fragments, those containing protospacer adjacent motif (PAM) sequences were selected, where the PAM sequence either starts with “TTTV” or ends with “VAAA”. The target species’ fragment sequences were then aligned against those of other species, and fragments exhibiting three or more mismatches as well as all indels (excluding PAM sequences) were retained to construct a species-specific target library for *Alternaria*. Finally, the occurrence frequencies and upstream and downstream sequences of these retained fragments were recorded.

#### 2.2.2. Selection of Reference Targets for Each Alternaria Species

For the 31 *Alternaria* species with available reference genomes, we performed additional sequence alignments using NCBI’s Basic Local Alignment Search Tool (BLAST, https://blast.ncbi.nlm.nih.gov/) for one target sequence from each species. The selection standard remained consistent, based on the 21 bp sequence differences excluding the PAM sequence, and the alignment information for the specific target sequences was recorded (Supplementary Figure S1).

### 2.3. Cultivation and DNA Extraction of Strains

This study used seven strains for experimental validation: *A. arbusti*, *A. infectoria*, *A. solani*, *A. tenuissima*, *A. triticina*, *A. alternata*, and *A. longipes*. The selection criteria were based on the availability of genomic information for the species, the commercial availability of the strains, and their high research value. These strains were obtained from the BeNa Culture Collection (BNCC, Henan, China), the Shanghai China General Microbiological Culture Collection Center (SHBCC, Shanghai, China), and the China General Microbiological Culture Collection Center (CGMCC, Beijing, China). The strains were cultured on a PDA medium, and a suitable amount of mycelium was ground in liquid nitrogen. Genomic DNA was extracted using a commercial DNA extraction kit (DP305, Tiangen Biotech, Beijing, China) according to the manufacturer’s instructions. DNA integrity and concentration were assessed by 1.5% agarose gel electrophoresis in 1× TAE buffer at 100 V for 45 min and by using a Nanodrop 2000 spectrophotometer (Thermo Fisher Scientific, Waltham, MA, USA).

### 2.4. Detection of Specific Targets Using Sanger Sequencing

Primers, 25 bp in length, targeting the 150 bp regions upstream and downstream of the target sequence, were designed using Primer Premier 6.0, with amplicon sizes ranging from 200 to 350 bp. The primers were synthesized by SinoGenoMax (SinoGenoMax Co. Ltd., Beijing, China). PCR amplification was carried out in a 25 μL reaction mixture, containing 12.5 µL of 2× Taq PCR MasterMix (Aidlab Biotechnologies Co. Ltd., Beijing, China), 8.5 µL of nuclease-free water, 2 µL of genomic DNA (10 ng/µL), and 1 µL each of forward and reverse primers (10 μmol/L). Amplification was carried out using an Applied Biosystems Veriti™ Thermal Cycler (Waltham, MA, USA) under the following conditions: an initial denaturation at 95 °C for 3 min, followed by 30 cycles of 95 °C for 30 s, 56 °C for 30 s, and 72 °C for 30 s, with a final extension at 72 °C for 10 min. PCR products were purified using the Universal DNA Purification Kit (DP214, Tiangen Biotech, Beijing, China), following the manufacturer’s instructions. The purified products were subjected to bidirectional Sanger sequencing on an ABI 3730 XL sequencer (Thermo Fisher Scientific, Waltham, MA, USA), and the resulting sequencing data were assembled using CodonCode Aligner (CodonCode Co., Dedham, MA, USA), with low-quality sequences removed.

### 2.5. Rapid Detection of Specific Targets Using CRISPR-Cas12a

The combination of enzymatic recombinase amplification (ERA) and CRISPR-Cas12a allowed for the rapid identification of specific targets. This process consisted of two main steps: amplification and detection. The amplification system used the Basic Nucleic Acid Amplification Kit (KS101, Gendx, Co. Ltd., Beijing, China) according to the instructions, with a template DNA concentration of 10 ng/µL at a volume of 2 µL. The total volume of the detection system was 46 µL, consisting of 30.7 µL of nuclease-free water, 10 µL of 10× NEBuffer 2.1 (New England Biolabs, (Beijing) Ltd., Beijing, China), and 3.3 µL of CRISPR RNA (crRNA), consisting of the universal nucleotide sequence UAAUUUCUACUAAGUGUAGAU and the target-specific sequence (excluding the PAM sequence), was obtained from Genscript (Genscript Co. Ltd., Nanjing, China), and 2 µL of Cas12a (20 nM, New England Biolabs, (Beijing) Ltd., China), incubated at 37 °C for 10 min. Then, 4 µL of ssDNA (/5′6-FAM/CCCCCCCCCC/3′BHQ-1, 400 nM, Suzhou Jinweizhi Biotechnology Co., Ltd., Suzhou, China) was added. Visual fluorescence and fluorescence intensity detection were performed using a blue light illuminator (BG-Vtrans520s, Baygene Biotech (Beijing) Co., Ltd., Beijing, China) and a fluorescence microplate reader (Thermo Fisher Co., Ltd., Waltham, MA, USA) at λ ex 483 nm/λ em 535 nm. The ssDNA was replaced with an 80 nM FAM-Biotin fluorescent signal molecule (/5′6-FAM/TTATTATT/3′Bio), and commercially available lateral flow strips (TS104, Suzhou Gendx Biotech Co., Ltd., Suzhou, China) were inserted into a centrifuge tube containing the sample to observe the results.

### 2.6. Statistical Analyses

*p*-values were calculated using the *t*-test (two groups). Differences with *p*-values *<* 0.05 were considered statistically significant. GraphPad Prism 8.0 was used for data analysis, graph generation, and statistical evaluation.

## 3. Results

### 3.1. Construction of a Species-Specific Target Library for Alternaria Species

#### 3.1.1. Main Factors Affecting the Number of Specific Target Sequences

**Figure 2:**
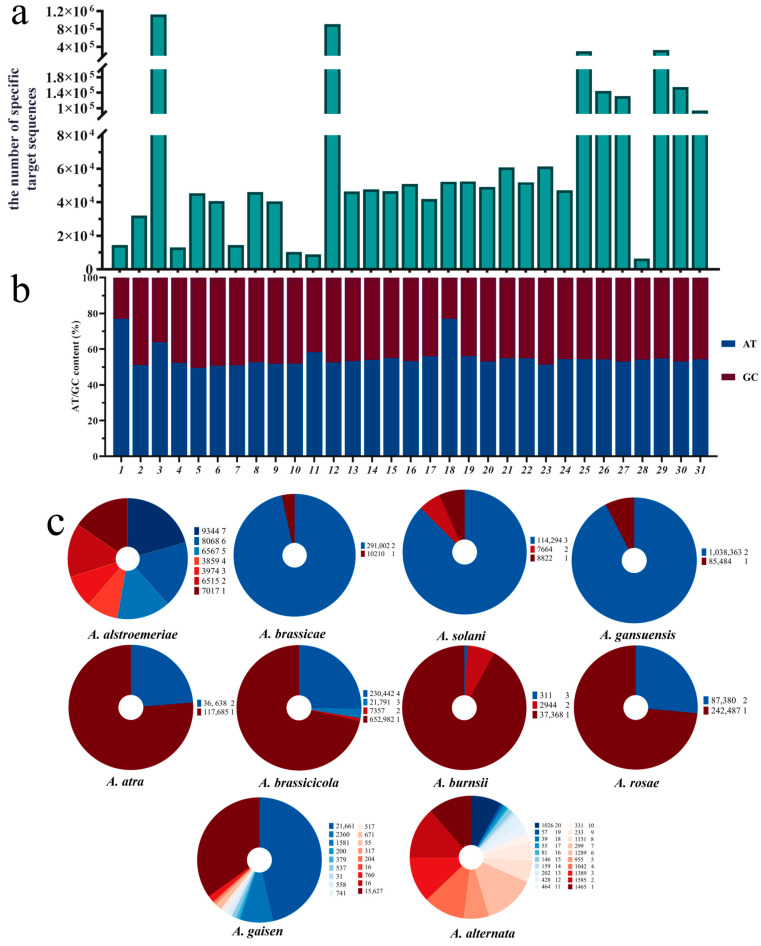
(**a**) The species-specific target sequence library of *Alternaria* species and their genome coverage based on reference genomes. 1: *A. alstroemeriae*; 2: *A. brassicae*; 3: *A. gansuensis*; 4: *A. alternata*; 5: *A. arborescens*; 6: *A. burnsii*; 7: *A. destruens*; 8: *A. gaisen*; 9: *A. longipes*; 10: *A. postmessia*; 11: *A. tenuissima*; 12: *A. brassicicola*; 13: *A. arbusti*; 14: *A. conjuncta*; 15: *A. ethzedia*; 16: *A. hordeiaustralica*; 17: *A. incomplexa*; 18: *A. infectoria*; 19: *A. metachromatica*; 20: *A. novae-zelandiae*; 21: *A. triticina*; 22: *A. triticimaculans*; 23: *A. ventricose*; 24: *A. viburni*; 25: *A. panax*; 26: *A. dauci*; 27: *A. solani*; 28: *A. porri*; 29: *A. rosae*; 30: *A. atra*; 31: *A. consortialis*. Numbers 4–11 belong to sect. *Alternaria*; 5 belongs to sect. *Brassicicola*; 12–24 belong to sect. *Brassicicola*; 25 belongs to sect. *Panax*; 26–28 belong to sect. *Porri*; 29 belongs to sect. *Pseudoalternaria*; and 30–31 belong to sect. *Ulocladioides*. (**b**) AT/CG content ratio of the species-specific target sequence library of 31 *Alternaria* species. (**c**) The distribution of specific target sequence quantities across the genomes of 10 species with two or more genomes. The color blocks in the legend correspond to the figures: the first column of the data set indicates the number of specific targets, while the second column shows the number of genomes containing shared specific targets.

We constructed specific-target sequence libraries for 31 *Alternaria* species, with the number of target sequences in each library ranging from 6385 to 1,123,847. The species with the highest number of target sequences contained more than 100 times the number of sequences found in the species with the fewest. The selected specific target sequences exhibited sufficient specificity within the *Alternaria* genus (Figure 2a).

Among the 31 *Alternaria* species genomes downloaded, ten species contained two or more genomes, and Figure 2c illustrates the distribution of target sequences in these multi-genome species. The results showed a decreasing trend in the number of shared target sequences as the number of genomes increased. However, there were still sufficient specific target sequences available for species identification and analysis.

In this study, reference-specific target sequences were selected for each of the 31 species. BLAST alignment analysis revealed that these target sequences exhibited significant nucleotide differences compared to those of other species, thereby meeting the predefined screening criteria. Additionally, for 10 of the species, the reference-specific target sequences exclusively matched their own sequences during the alignment process, achieving 100% sequence identity (Supplementary Figure S1).

#### 3.1.2. GC Content Distribution in the Species-Specific Target Library

Furthermore, we calculated the GC content of each species’ target sequence library (sequences excluding the PAM region) (Figure 2b). The results showed that the GC content of 28 species ranged from 40% to 51%, accounting for more than 90% of the total, indicating that these target sequences possessed favorable thermal stability and experimental feasibility. This GC content range also contributed to improved amplification efficiency and reduced non-specific binding. Although the overall GC content of the species-specific target library for some species fell below this range—such as *A. gansuensis* with a GC content of 36.10%, and *A. alstroemeriae* and *A. destruens* with GC contents of 23.06% and 23.03%, respectively—target sequences with GC content between 40% and 60% were still found in the specific target library of these species. Therefore, even with lower GC content, it was still possible to select target sequences with suitable GC content to meet experimental requirements.

#### 3.1.3. The Distribution Characteristics of Specific-Target Sequences Across the Whole Genome

Based on the AGE principle, we used BLAST analysis on NCBI to successfully select reference-specific target sequences for each of the 31 species from the species-specific target sequence library. We also obtained the corresponding chromosomal positions, annotation details, and sequence locations (Table 1 and Table S2). Among the reference-specific target sequences of these 31 species, 9.68% had clear chromosomal position assembly, 41.94% had been annotated, and 9.68% were identified as protein-coding. The target sequences were widely distributed, ranging from positions 34–58 to 2,381,784–2,381,808, with a relatively random distribution. Furthermore, even in the three species with a GC content below 40% (*A. alstroemeriae*, *A. destruens*, and *A. gansuensis*), suitable target sequences were still identified, with GC contents of 47.62%, 42.86%, and 42.86%, respectively. Through this screening and analysis, we successfully identified target sequences that meet the specificity requirements for all 31 species of *Alternaria*.

### 3.2. Detection of Target Species Using Sanger Sequencing

**Figure 3:**
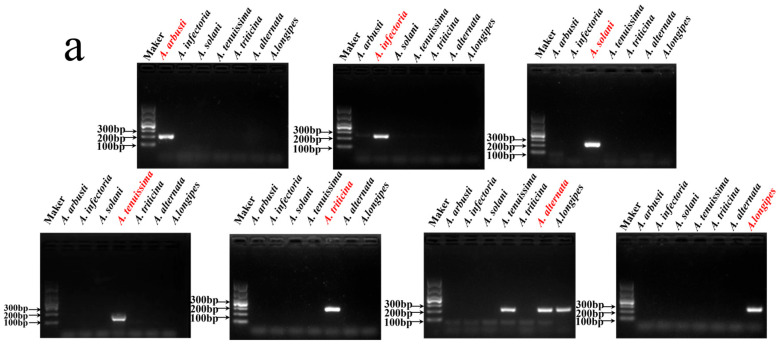

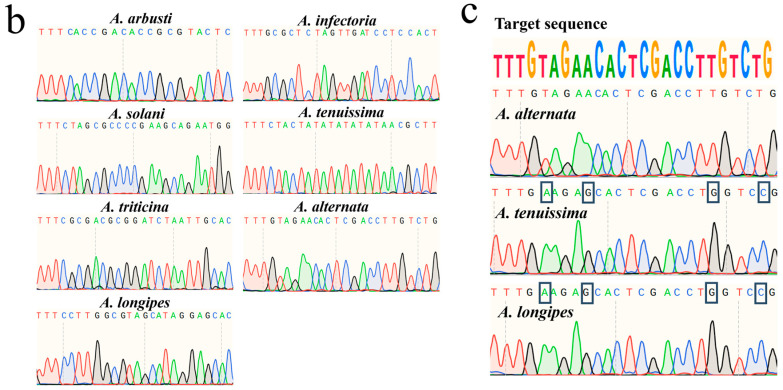
(**a**) On the same agarose gel electrophoresis image, primers to the red-marked species are used for PCR amplification of DNA from seven species.(**b**) Sequencing results for the seven target species. (**c**) *A. alternata* primer amplification sequencing peak alignment, square boxes indicate bases that differ when aligned with the target sequence of *A. alternata*.

To assess the practical applicability of the selected specific target sequences, we experimentally validated them using seven species. Primers were designed for each of these species, and PCR amplification was performed (Supplementary Table S3). Agarose gel electrophoresis results showed clear bands in the 100–300 bp range for all target species (Figure 3a), indicating that the designed primers effectively amplified the specific regions of each target species. Notably, due to the closely related species between *A. tenuissima*, *A. alternata*, and *A. longipes*, these species also exhibited similar bands in the PCR amplification with *A. alternata* primers. To further confirm the accuracy of the PCR products, we performed Sanger sequencing of the amplified products from all target species. The results showed that all amplification products contained the expected target sequences (Figure 3b). Specifically, the sequences amplified with *A. alternata* primers showed four base pair differences when compared to *A. tenuissima* and *A. longipes* (Figure 3c), providing preliminary validation of the specificity of the selected target sequences. Overall, these experimental results demonstrate that, except for the primer pair targeting *A. alternata*, the remaining six synthesized primer pairs exhibit high specificity among the seven experimentally validated species. The specific target sequences of each species are exclusively present within their respective species. Moreover, this method enables precise species identification based on minor sequence differences, effectively distinguishing even closely related species at the genomic level.

### 3.3. Detection of Target Species Using the CRISPR-Cas12a Dual System

Compared to traditional methods, ERA and CRISPR-Cas12a allow for rapid and accurate species identification in as little as 30 min. In this study, ERA was used for isothermal amplification, and CRISPR-Cas12a was paired with synthetic crRNAs targeting species-specific sequences (Supplementary Table S4). Two detection systems were employed: a fluorescence system and a lateral flow strip assay, which mainly differ in CRISPR-Cas12a probe selection and incubation time.

#### 3.3.1. Sensitivity Experiment of the CRISPR-Cas12a Dual System

**Figure 4:**
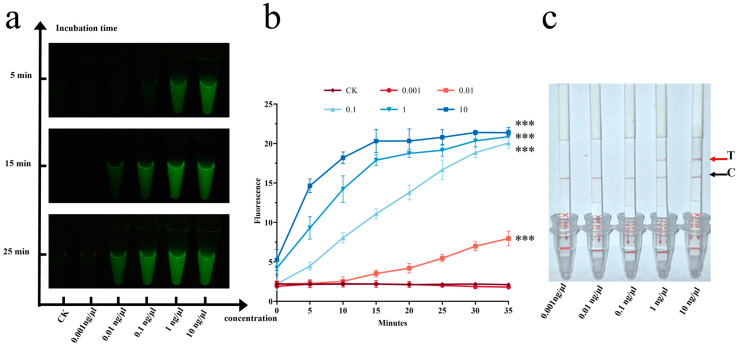
(**a**) Fluorescence intensity of *A. alternata* at concentrations of 0.001, 0.01, 0.1, 1, and 10 ng/μL after incubation for 5, 15, and 25 min. (**b**) Microplate reader values of *A. alternata* at the same concentrations after 35 min of incubation. *** *p* < 0.001. (**c**) Lateral flow strip assay results for *A. alternata* at the same concentrations; T: test line; C: control line.

*A. alternata* was selected for preliminary experiments. Sensitivity testing revealed that samples with concentrations of 1 ng/μL and 10 ng/μL exhibited strong fluorescence immediately after the addition of ssDNA. With extended incubation, samples at concentrations of 0.1 ng/μL and 0.01 ng/μL also began to show fluorescence (Figure 4a).

Statistical analysis using the *t*-test showed consistent results. After 25 min of incubation, samples at concentrations of 0.01 ng/μL, 0.1 ng/μL, 1 ng/μL, and 10 ng/μL showed highly significant differences (*p* < 0.001) compared to the control group (CK), while no significant difference was observed for the 0.001 ng/μL samples (Figure 4b). These results indicated that the detection limit of the fluorescence system is 0.01 ng/μL.

In contrast, the lateral flow strip assay had a detection limit of 1 ng/μL, with the most prominent bands observed at 10 ng/μL (Figure 4c). This suggests that the fluorescence system is significantly more sensitive than the lateral flow assay, which is consistent with findings reported in previous studies [48]. Therefore, subsequent experiments used a sample concentration of 10 ng/μL.

#### 3.3.2. Specificity Experiment of the CRISPR-Cas12a Dual System

**Figure 5:**
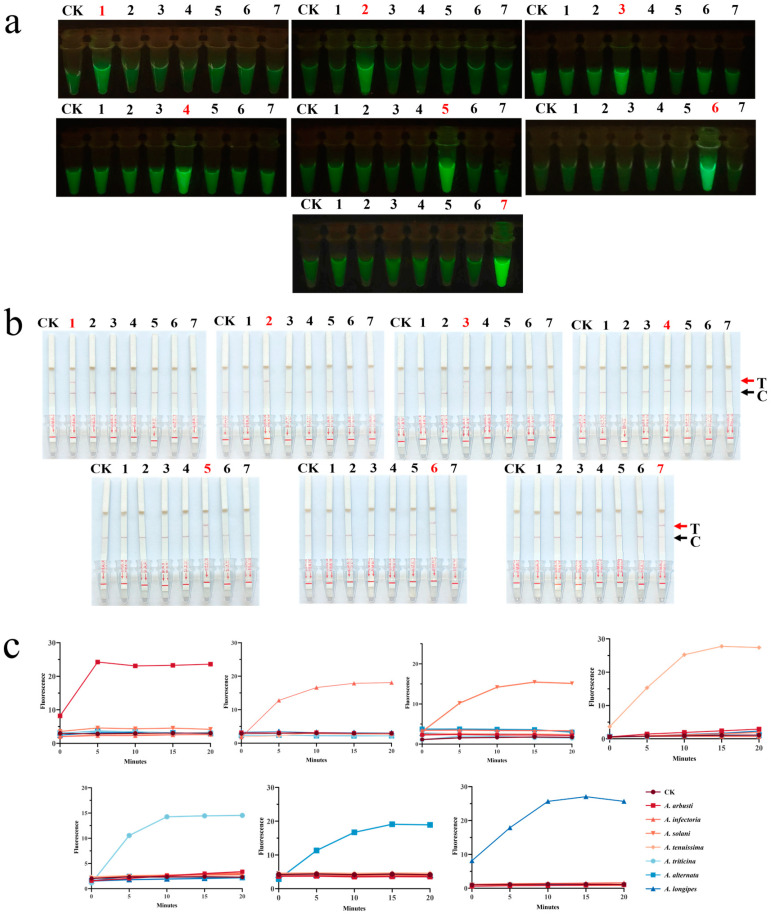
(**a**–**c**) Specificity experiments were performed for the seven target species using the seven selected primers and crRNAs. The CRISPR-Cas12a dual-system, combined with microplate reader, visible fluorescence, and lateral flow strip assays, successfully achieved precise identification of each species, with no cross-reaction observed with non-target species in any of the experiments. CK: nuclease-free water used as a negative control; 1: *A. arbusti*; 2: *A. infectoria*; 3: *A. solani*; 4: *A. tenuissima*; 5: *A. triticina*; 6: *A. alternata*; 7: *A. longipes.* In the same picture, primers to the red-marked species are used to amplify DNA from seven species.

To further assess the specificity of the CRISPR-Cas12a dual system, we tested *A. arbusti*, *A. infectoria*, *A. solani*, *A. tenuissima*, *A. triticina*, *A. alternata*, and *A. longipes* using both the fluorescence system and the lateral flow strip assay. The results of the CRISPR-Cas12a fluorescence system were presented in two ways: fluorescence values and visible fluorescence. Microplate reader measurements showed that the fluorescence intensity for the target species stabilized after approximately 10 min. The fluorescence values varied significantly across the seven species, likely due to differences in primer amplification efficiency [45]. Blue light exposure revealed strong fluorescence reactions only for the target species (Figure 5a,b). The results from the CRISPR-Cas12a lateral flow system were consistent with those of the fluorescence system, with positive results observed only in samples containing the target species (Figure 5c). The experimental results demonstrated that the CRISPR-Cas12a dual system can specifically identify the target species and yield consistent detection results across different species, exhibiting excellent specificity.

## 4. Discussion

### 4.1. AGE Enables the Construction of a Species-Specific Target Library for Alternaria Genus Identification

From the perspective of AGE analysis, the specific target sequences we select are distributed across various regions of the entire genome. Therefore, in species identification, for strains with complete DNA information, we can randomly choose a specific target sequence for identification, which typically yields accurate results.

Through bioinformatics analysis, we constructed a species-specific target sequence database for each species within the genus *Alternaria*. By selecting reference-specific target sequences from each species and performing BLAST alignment, the results showed that each species possesses a unique reference target sequence. These target sequences exhibit a high degree of specificity across species, facilitating accurate species identification. The number of species-specific target sequences in the database is influenced by three main factors.

Firstly, the scope of species included in the analysis also significantly affects the number of target sequences. In this study, we constructed a target sequence database based on the genomic data of 31 species within the genus *Alternaria*, covering all known genomes of the species in this genus. In contrast, the fungal genus *Penicillium* selected only seven species’ genomes to build its target sequence database [46]. Due to the differences in the range of species selected, the number of species-specific target sequences for *Alternaria* is significantly lower. This suggests that the more species included in the analysis, the fewer the species-specific target sequences, which in turn helps improve the accuracy of species identification.

Secondly, the phylogenetic relationship between species is also an important factor influencing the number of target sequences. Closely related species tend to share more genetic information and structural similarities in their genomes, which results in fewer species-specific target sequences between them [49]. For example, *A. alternata* and *A. tenuissima* are closely related species, and due to their high similarity in genome structure and genetic background, it becomes challenging to effectively distinguish between them, leading to a relatively low number of species-specific target sequences. In summary, the number of species-specific target sequences in the target sequence database is influenced by multiple factors, including the scope of species included in the analysis, and the phylogenetic relationships between species.

Intraspecific variation, although significantly lower than interspecific variation, plays a crucial role in the selection of specific target sequences for accurate identification [50]. A notable exception is *A. alternata*. In this study, a total of 81 *A. alternata* genomes were used, of which 61 were artificially mutated. The analysis indicates that these artificial mutations may lead to the loss or alteration of target sequences, resulting in only a few common target sequences between the mutated and wild-type *A. alternata* [51]. These sequences are insufficient to cover all mutated genomes. Therefore, the *A. alternata* specific target sequences selected in this study are primarily applicable to wild-type *A. alternata* and strains with incomplete mutations.

### 4.2. AGE Demonstrates Accurate Species Identification Through Multiple Experimental Methods

Based on the AGE, successfully screened species-specific target sequences for *Alternaria* species, which can be validated through various techniques such as agarose gel electrophoresis, Sanger sequencing, and the CRISPR-Cas12a system. Compared with traditional identification methods, this study overcomes the limitations of morphological identification in distinguishing closely related species [29], addresses the insufficiency of ITS as a universal barcode in differentiating intraspecific variations [52], and reduces the complexity and high cost associated with multi-gene phylogenetic analysis [53]. This study employs agarose gel electrophoresis for specificity amplification, although with relatively lower accuracy. The initial equipment investment for this method is around USD 300, with reagent costs approximately USD 4.97 per sample [54]. Sanger sequencing is used to validate specific target sequences selected by AGE. As the gold standard for species identification [54], despite the higher costs of equipment purchase and maintenance, Sanger sequencing offers reagent costs of USD 9.17 per sample [55], providing a precise validation method [56]. The CRISPR-Cas12 system is renowned for its high sensitivity in accurately detecting DNA from target species [57]. This system requires minimal initial equipment investment, with items like the Molecular Fluorescence Viewer priced at only USD 28 and reagent costs around USD 13.78 per sample [50], making it an efficient and convenient option. This method is highly valuable for agricultural applications, facilitating early pathogen identification, allowing for timely implementation of disease control measures, effectively reducing the spread of plant diseases, and mitigating economic losses caused by infections [58]. Based on experimental requirements, multiple detection methods are available for selection. However, these methods rely on existing genomic data, which may limit their scope of application.

### 4.3. AGE Enables Identification of Key Alternaria Species

Through AGE, we successfully screened a specific target sequence for seven species. When using *A. alternata* primers for PCR amplification and agarose gel electrophoresis of *A. alternata*, *A. tenuissima*, and *A. longipes*, all of which belong to sect. *Alternaria*, bands of the same size were observed. Studies have shown that species within sect. *Alternaria* exhibit genome similarities ranging from 96.7% to 98.2%, further confirming the high sequence similarity among these three species [29]. Subsequently, we successfully identified these closely related species within the same section using Sanger sequencing, effectively resolving the challenge of distinguishing them even with multi-gene sequence analysis [35,59,60]. These results indicate that AGE not only enables precise differentiation of closely related species within the same group but also provides valuable tools and references for mycologists and taxonomists.

It is well known that, according to the International Plant Protection Convention (IPPC), *A. triticinais* is a significant plant pathogen with high pathogenicity [61]. Due to the challenges associated with its morphological identification, quarantine efforts are further complicated. Notably, this study successfully identified a specific target sequence for *A. triticina* and implemented a CRISPR-Cas12a-based method for its rapid on-site detection. This achievement holds significant practical value in enhancing quarantine efficiency and strengthening the monitoring and control of *A. triticina* dissemination.

### 4.4. AGE: Challenges and Developments

Although we have validated the specificity of seven *Alternaria* species through various experimental methods, and the results indicated that AGE significantly improves species identification accuracy, further efforts are needed to expand its application scope and enhance reliability. Future research should include a broader range of samples, covering more species and samples from different ecological environments. It is important to note that *Alternaria* conidia may be present in soil, the atmosphere, plants, or indoor environments [62]. Therefore, avoiding false positives during field testing is crucial.

Although AGE holds great potential for species identification, species identification based on existing data still faces several challenges and limitations. First, the reliability of the species-specific target library heavily depends on the quality of the reference genome data. As of 30 December 2024, the NCBI database contains data for 423 *Alternaria* species, of which only 35 species have genome data, only 16 provide only a single genome, and 24 genomes allow identification only at the genus level. This situation indicates that the existing genome data still have significant limitations. In the future, stringent genome selection standards can be established to ensure that the genome data included in the analysis are highly reliable and representative [63]. Furthermore, as more species genome data are updated and improved, the accuracy and broad applicability of the specific target sequence library will be greatly enhanced.

Secondly, selecting appropriate specific target sequences is crucial for species identification. As shown in the analysis results of Figure 2c, some species exhibit significant intraspecific variation, suggesting that certain specific target sequences from the selected reference genome may not match the actual samples. Therefore, future research could explore and establish a more scientific principle for selecting specific target sequences [64], aiming to minimize the impact of intraspecific variation and thus improve the accuracy and reliability of species identification.

Finally, the accuracy and screening efficiency of the species-specific target library improve with the increase in the number of species covered. In this study, the specific target sequence library we established is limited to *Alternaria* species with genome data. To ensure that the selected specific target sequences are specific across all species, we also performed a BLAST comparison with the NCBI database. With the continuous accumulation and improvement of data, there is potential to develop an AGE data-sharing platform for species identification in the future [65]. Data sharing and standardization will significantly enhance the efficiency of specific target sequence screening and reduce errors associated with manual operations.

## 5. Conclusions

In this study, we employed bioinformatics methods to construct a target sequence library for species within the *Alternaria* genus and used various experimental techniques, including agarose gel electrophoresis, Sanger sequencing, and the CRISPR-Cas12a dual system, to validate the high specificity of the selected target sequences and successfully achieve accurate species-level identification. Moreover, for closely related *Alternaria* species, such as *A. tenuissima* and *A. alternata*, the species-specific target library demonstrated the ability to precisely differentiate them, overcoming the limitations of traditional species identification methods when dealing with closely similar species. When applied to complex environmental samples, the AGE has promise as a rapid, convenient, and efficient tool for species identification, with significant potential for widespread application.

## Figures and Tables

**Table 1:** Reference-specific target sequences and annotations for each *Alternaria* species.

Species	Reference Target Sequence	Chromosomal Location	Annotation	Sequence Range
*A. arbusti*	TTTCACCGACACCGCGTACTCTGTC	no assembled	/	1,506,110–1,506,134
*A. infectoria*	TTTGCGCTCTAGTTGATCCTCCACT	no assembled	/	897,690–897,714
*A. solani*	TTTCTAGCGCCCCGAAGCAGAATGG	chromosome 2	/	632,284–632,308
*A. tenuissima*	TTTCTACTATATATATATAACGCTT	no assembled	O-methyltransferase	825–849
*A. triticina*	TTTCGCGACGCGGATCTAATTGCAC	no assembled	/	62,527–62,551
*A. alternata*	TTTGTAGAACACTCGACCTTGTCTG	no assembled	Nnf1-domain-containing protein	370,978–371,002
*A. longipes*	TTTCCTTGGCGTAGCATAGGAGCAC	chromosome 3	/	554,291–554,315

”/” indicates that there is no relevant annotation information in NCBI.

## Data Availability

All data supporting the findings of this study are available within the paper and within its Supplementary Materials published online. Further inquiries can be directed at the corresponding author.

## Supplementary Materials

The following supporting information can be downloaded at https://www.mdpi.com/article/10.3390/jof11030185/s1: Table S1: Reference genome data table for *Alternaria*; Table S2: Reference-specific target sequences and annotations for each *Alternaria* species; Table S3: Primers for the seven species used in experimental validation; Table S4: crRNA for the seven species used in experimental validation; Figure S1: Screening process of reference-specific target sequences.

## References

[1] DeMers M. (2022). *Alternaria alternata* as endophyte and pathogen. Microbiology.

[2] Li J.F., Jiang H.B., Jeewon R., Hongsanan S., Bhat D.J., Tang S.M., Lumyong S., Mortimer P.E., Xu J.C., Camporesi E. (2023). *Alternaria*: Update on species limits, evolution, multi-locus phylogeny, and classification. Stud. Fungi.

[3] Wijayawardene N.N., Hyde K.D., Al-Ani L.K.T., Tedersoo L., Haelewaters D., Rajeshkumar K.C., Zhao R., Aptroot A., Leontyev D., Saxena R. (2020). Outline of Fungi and fungus-like taxa. Fungal Biol..

[4] Agriopoulou S., Stamatelopoulou E., Varzakas T. (2020). Advances in occurrence, importance, and mycotoxin control strategies: Prevention and detoxification in foods. Foods.

[5] Stengel A., Stanke K.M., Quattrone A.C., Herr J.R. (2022). Improving taxonomic delimitation of fungal species in the age of genomics and phenomics. Front. Microbiol..

[6] Wang J., Zhai W., Gao H., Han R., Qi F. (2017). Serious damage to crop production caused by *Alternaria* diseases and the safety of agricultural products. Plant Prot..

[7] Kang Z., Jiang L., Luo Y., Liu C., Li X. (2013). The research advances of mechanism of pathogenicity of *Alternaria* phytopathogenic fungi. Chin. Bull. Life Sci..

[8] Feng Z., Li Y., Ma X., Duan Y., Zhang R., Hsiang T., Niu Y., Sun G. (2022). Draft Genome Sequence of *Alternaria longipes* Causing Tobacco Brown Spot. Plant Dis..

[9] Chen Y.-h., Wu Y.Z., Liu Q., Xia Z., Wang J., Luo X.X. (2023). Streptomyces tamarix sp. nov.: Antagonism against *Alternaria gaisen* producing streptochlorin, isolated from Tamarix root soil. Front. Microbiol..

[10] Al Lami H.F., You M.P., Barbetti M.J. (2020). Temperature Drives Contrasting *Alternaria* Leaf Spot Epidemic Development in Canola and Mustard Rape from *Alternaria japonica* and *A. brassicae*. Plant Dis..

[11] Zhang Y., Zhang X.L., Wang C., Luo M., Hai Bo M.A., Zhang Y. (2015). Detection of *Alternaria triticina* Prasada & Prabhu using PCR techniques. Biosafety.

[12] Tozlu E., Tekiner N., Kotan R., Örtücü S. (2018). Investigation on the biological control of *Alternaria alternata*. Indian J. Agric. Sci..

[13] Fan Q., Zhou Q., Zhang S., Li Y., Li J., Chen X., Sun L. (2024). First Report of Leaf Spot Disease Caused by *Alternaria alternata* on *Polygonatum cyrtonema* in Hunan Province of China. Plant Dis..

[14] Wang W., Zhao Y.Q., Chen Y., Zhang S.J., Zhu C.C. (2024). First Report of Leaf Spot on Chaste Tree Caused by *Alternaria alternata* in China. Plant Dis..

[15] Qi H., Li Z., Xu T., Lu G., Wang X., Ma M., Ni R., Shen B., Chang J. (2024). First Report of Leaf Spot on *Taraxacum mongolicum* Caused by *Alternaria solani* in China. Plant Dis..

[16] Lee H.B., Patriarca A., Magan N. (2015). *Alternaria* in food: Ecophysiology, mycotoxin production and toxicology. Mycobiology.

[17] Ali S., Tyagi A., Rajarammohan S., Mir Z.A., Bae H. (2023). Revisiting *Alternaria*-host interactions: New insights on its pathogenesis, defense mechanisms and control strategies. Sci. Hortic..

[18] Dalinova A., Salimova D., Berestetskiy A. (2020). Fungi of the genera *Alternaria* as producers of biological active compounds and mycoherbicides. Appl. Biochem. Microbiol..

[19] Zhao S., Li J., Liu J., Xiao S., Yang S., Mei J., Ren M., Wu S., Zhang H., Yang X. (2023). Secondary metabolites of *Alternaria*: A comprehensive review of chemical diversity and pharmacological properties. Front. Microbiol..

[20] Zhao L., Luo H., Cheng H., Gou Y.N., Yu Z.H., Deng J.X. (2022). New species of large-Spored *Alternaria* in section *Porri* associated with Compositae plants in China. Fungi.

[21] Iturrieta-González I., Gené J. (2023). *Alternaria muriformis* sp. nov., a New Species in Section *Chalastospora* Isolated from Herbivore Dung in Spain. Diversity.

[22] Oviedo M.S., Sturm M.E., Reynoso M.M., Chulze S.N., Ramirez M.L. (2013). Toxigenic profile and AFLP variability of *Alternaria alternata* and *Alternaria infectoria* occurring on wheat. Braz. J. Microbiol..

[23] Elfar K., Bustamante M.I., Arreguin M., Nouri M.T., Eskalen A. (2023). Identification and pathogenicity of *Alternaria* species causing leaf blotch and fruit spot of apple in California. Phytopathol. Mediterr..

[24] Meyer W., Irinyi L., Hoang M.T.V., Robert V., Garcia-Hermoso D., Desnos-Ollivier M., Yurayart C., Tsang C.-C., Lee C.-Y., Woo P.C. (2019). Database establishment for the secondary fungal DNA barcode translational elongation factor 1α (TEF1α). Genome.

[25] Aylagas E., Borja Á., Irigoien X., Rodríguez-Ezpeleta N. (2016). Benchmarking DNA metabarcoding for biodiversity-based monitoring and assessment. Front. Mar. Sci..

[26] Laich F.S., Alcoba Flórez J., Pérez Roth E., Bahaya Y., Luis Delgado J., Méndez Álvarez S. (2008). Molecular characterization of *Alternaria alternata* causing ocular infection: Detection of IGS-RFLP intraspecific polymorphism. Sabouraudia.

[27] Lücking R., Aime M.C., Robbertse B., Miller A.N., Ariyawansa H.A., Aoki T., Cardinali G., Crous P.W., Druzhinina I.S., Geiser D.M. (2020). Unambiguous identification of fungi: Where do we stand and how accurate and precise is fungal DNA barcoding?. IMA Fungus.

[28] Roberts R., Reymond S., Andersen B. (2000). RAPD fragment pattern analysis and morphological segregation of small-spored *Alternaria* species and species groups. Mycol. Res..

[29] Woudenberg J., Seidl M., Groenewald J., De Vries M., Stielow J., Thomma B., Crous P. (2015). *Alternaria* section *Alternaria*: Species, formae speciales or pathotypes?. Stud. Mycol..

[30] Andersen B., Krøger E., Roberts R.G. (2002). Chemical and morphological segregation of *Alternaria arborescens*, *A. infectoria* and *A. tenuissima* species-groups. Mycol. Res..

[31] Zwickel T., Kahl S.M., Rychlik M., Müller M.E. (2018). Chemotaxonomy of mycotoxigenic small-spored *Alternaria* fungi–do multitoxin mixtures act as an indicator for species differentiation?. Front. Microbiol..

[32] Andrew M., Peever T., Pryor B. (2009). An expanded multilocus phylogeny does not resolve morphological species within the small-spored *Alternaria* species complex. Mycologia.

[33] De Hoog G., Horré R. (2002). Molecular taxonomy of the *Alternaria* and *Ulocladium* species from humans and their identification in the routine laboratory. Mycoses.

[34] Nichea M.J., Cendoya E., Romero C.J., Humaran J.F., Zachetti V.G., Palacios S.A., Ramirez M.L. (2022). Phylogenetic analysis and toxigenic profile of *Alternaria* species isolated from chickpeas (*Cicer arietinum*) in Argentina. Diversity.

[35] Ramires F.A., Masiello M., Somma S., Villani A., Susca A., Logrieco A.F., Luz C., Meca G., Moretti A. (2018). Phylogeny and mycotoxin characterization of *Alternaria* species isolated from wheat grown in Tuscany, Italy. Toxins.

[36] Yu X., Zhang J., Zhang X., Yang X., Xu X., Lin J., Bing H., Wang X., Zhao J., Xiang W. (2022). Identification and pathogenicity of fungi associated with leaf spot of muskmelon in eastern Shandong Province, China. Plant Dis..

[37] Xu X., Zhang L., Yang X., Cao H., Li J., Cao P., Guo L., Wang X., Zhao J., Xiang W. (2022). *Alternaria* spp. associated with leaf blight of maize in Heilongjiang Province, China. Plant Dis..

[38] Falcon R.M.G., Sumabat Dacones L.G. (2022). Phylogeography of Fungicide Resistance of *Alternaria alternata* and *Alternaria tenuissima* to Demethylation Inhibitor Fungicides. Sci. Diliman.

[39] He L., Cheng H., Zhao L., Htun A.A., Yu Z.H., Deng J.X., Li Q.L. (2021). Morphological and molecular identification of two new *Alternaria* species (Ascomycota, Pleosporaceae) in section Radicina from China. MycoKeys.

[40] Gao L., Xu W., Xin T., Song J. (2023). Application of third-generation sequencing to herbal genomics. Front. Plant Sci..

[41] Xin T., Zhang Y., Pu X., Gao R., Xu Z., Song J. (2019). Trends in herbgenomics. Sci. China Life Sci..

[42] Gan Y., Xin T., Xu W., Hao L., Qi G., Lou Q., Song J. (2023). Principles and Strategies for Species Identification Based on Analysis of whole-Genome. Acta Pharm. Sin..

[43] Gan Y., Qi G., Hao L., Xin T., Lou Q., Xu W., Song J. (2024). Analysis of Whole-Genome as a Novel Strategy for Animal Species Identification. Int. J. Mol. Sci..

[44] Hao L., Xu W., Qi G., Xin T., Xu Z., Lei H., Song J. (2022). GAGE is a method for identification of plant species based on whole genome analysis and genome editing. Commun. Biol..

[45] Qi G., Hao L., Gan Y., Xin T., Lou Q., Xu W., Song J. (2024). Identification of closely related species in *Aspergillus* through Analysis of Whole-Genome. Front. Microbiol..

[46] Huang Y., Fu L., Gan Y., Qi G., Hao L., Xin T., Xu W., Song J. (2024). Analysis of Whole-Genome for Identification of Seven *Penicillium* Species with Significant Economic Value. Int. J. Mol. Sci..

[47] Qi G., Hao L., Xin T., Gan Y., Lou Q., Xu W., Song J. (2024). Analysis of whole-Genome facilitates rapid and precise identification of fungal species. Front. Microbiol..

[48] Deng Z., Hu H., Tang D., Liang J., Su X., Jiang T., Hu X., Ying W., Zhen D., Xiao X. (2022). Ultrasensitive, Specific, and Rapid Detection of *Mycoplasma pneumoniae* Using the ERA/CRISPR–Cas12a Dual System. Front. Microbiol..

[49] Zi Ming C., Yi Feng C. (2000). Genetic relationships of the specialized schizothoracine fishes inferred from random amplified polymorphic dna analysis. Zool. Res..

[50] He X.-L., Li Q., Peng W.-H., Zhou J., Cao X.-L., Wang D., Huang Z.-Q., Tan W., Li Y., Gan B.-C. (2017). Intra-and inter-isolate variation of ribosomal and protein-coding genes in Pleurotus: Implications for molecular identification and phylogeny on fungal groups. BMC Microbiol..

[51] Paterson R.R.M., Lima N. (2013). Biochemical mutagens affect the preservation of fungi and biodiversity estimations. Appl. Microbiol. Biotechnol..

[52] Marin-Felix Y., Hernández-Restrepo M., Iturrieta-González I., García D., Gené J., Groenewald J.Z., Cai L., Chen Q., Quaedvlieg W., Schumacher R. (2019). Genera of phytopathogenic fungi: GOPHY 3. Stud. Mycol..

[53] Nwe Z.M., Htut K.N., Aung S.L.L., Gou Y.-N., Huang C.-X., Deng J.-X. (2024). Two novel species and a new host record of *Alternaria* (Pleosporales, Pleosporaceae) from sunflower (Compositae) in Myanmar. MycoKeys.

[54] Rybnicky G.A., Dixon R.A., Kuhn R.M., Karim A.S., Jewett M.C. (2022). Development of a freeze-dried CRISPR-Cas12 sensor for detecting wolbachia in the secondary science classroom. ACS Synth. Biol..

[55] Nie Z., Lü P., Zhang R., Tu Y., Liu Z., Li Y., Tang C., Li X., Zhao K., Zhou Q. (2021). A simple and rapid method for fish sex identification based on recombinase-aided amplification and its use in Cynoglossus semilaevis. Sci. Rep..

[56] Cheng C., Fei Z., Xiao P. (2023). Methods to improve the accuracy of next-generation sequencing. Front. Bioeng. Biotechnol..

[57] Swarts D.C. (2019). Making the cut (s): How Cas12a cleaves target and non-target DNA. Biochem. Soc. Trans..

[58] Pan X., Zhou Y., Liu D., Wang Y., Sheng Y., Zhang H. (2024). Leaf Spot caused by *Alternaria tenuissima* on Rhamnella franguloides in China. Plant Dis..

[59] Woudenberg J., Groenewald J., Binder M., Crous P. (2013). *Alternaria* redefined. Stud. Mycol..

[60] Tan G., Yang Z., Yuan Z., Zhang S. (2013). Morphological, molecular and pathogenic characterization of *Alternaria longipes*, the fungal pathogen causing leaf spot on *Atractylodes macrocephala*. Microbiol. Res..

[61] Masiello M., El Ghorayeb R., Somma S., Carine S., Giuseppe M., Logrieco A.F., Habib W., Moretti A. (2022). *Alternaria* species and related mycotoxin detection in Lebanese durum wheat grain. Phytopathol. Mediterr..

[62] Sánchez P., Vélez-del-Burgo A., Suñén E., Martínez J., Postigo I. (2022). Fungal allergen and mold allergy diagnosis: Role and relevance of *Alternaria alternata* Alt a 1 protein family. J. Fungi.

[63] Zeng Q., Cao W., Xing L., Qin G., Wu J., Nagle M.F., Xiong Q., Chen J., Yang L., Bajaj P. (2020). A novel high-accuracy genome assembly method utilizing a high-throughput workflow. bioRxiv.

[64] Ciufo S., Kannan S., Sharma S., Badretdin A., Clark K., Turner S., Brover S., Schoch C.L., Kimchi A., DiCuccio M. (2018). Using average nucleotide identity to improve taxonomic assignments in prokaryotic genomes at the NCBI. Int. J. Syst. Evol. Microbiol..

[65] McKenna A., Hanna M., Banks E., Sivachenko A., Cibulskis K., Kernytsky A., Garimella K., Altshuler D., Gabriel S., Daly M. (2010). The Genome Analysis Toolkit: A MapReduce framework for analyzing next-generation DNA sequencing data. Genome Res..

