# Supplementary material for: Analysis of Whole-Genome for Alternaria Species Identification
Source: J Fungi (Basel). 2025 Feb 26;11(3):185. doi: 10.3390/jof11030185 (PMC11943077; doi:10.3390/jof11030185)
Supplement: Supplementary file 1 [file jof-11-00185-s001.zip › jof-3459704-supplementary.pdf]

Table S1. Reference Genome Data Table for *Alternaria.*

Species	Strain	GenBank	Submitter
Alternaria alstroemeriae	LH_A124	GCA_037044435.1	Qingdao Agricultural University
Alternaria alternata	ATCC 34957	GCA_001443195.1	Agriculture and Agri-Food Canada
	Z7	GCA_001572055.1	Zhejiang University
	SRC1lrK2f	GCA_001642055.1	DOE Joint Genome Institute
	MOD1-FUNGI5	GCA_004634295.1	USFDA
	JS-1623	GCA_009650635.1	National Institute of Biological Resources
	PN1	GCA_011420445.1	
	JS-0527	GCA_011420255.1	
	PN2	GCA_011420565.1	
	B3	GCA_014154925.1	Hangzhou Normal University
	Z7	GCA_014751505.1	Zhejiang University
	PF1	GCA_018104255.1	The Institute of Oceanology, Chinese Academy of Sciences
	Y784-BC03	GCA_020085065.1	Chongqing University of Arts & Sciences
	hznu325	GCA_020449105.1	Hangzhou Normal University
	MPI-PUGE-AT-0064	GCA_020736535.1	DOE Joint Genome Institute
	DET2010	GCA_020736745.1	Agriculture and Agri-Food Canada
	KAS5516	GCA_020736765.1	
	DET2001	GCA_020736775.1	
	Y17	GCA_022344125.1	Beijing Forestry University
	Y16	GCA_022344135.1	
	Y3	GCA_022344145.1	
	Y4	GCA_022344155.1	
	Y2	GCA_022344165.1	
	Y15	GCA_022344205.1	
	Y13	GCA_022344225.1	
	Y14	GCA_022344235.1	
	Y12	GCA_022344255.1	
	Y10	GCA_022344265.1	
	X9	GCA_022344295.1	
	Y1	GCA_022344325.1	
	X8	GCA_022344335.1	
	X7	GCA_022344355.1	
	X6	GCA_022344365.1	
	X16	GCA_022344405.1	
	X15	GCA_022344415.1	
	X12	GCA_022344435.1	
	X11	GCA_022344465.1	

	X1	GCA_022344485.1	
	H23	GCA_022344495.1	
	H3	GCA_022344505.1	
	H19	GCA_022344545.1	
	H20	GCA_022344555.1	
	H18	GCA_022344575.1	
	H15	GCA_022344615.1	
	H13	GCA_022344625.1	
	H11	GCA_022344635.1	
	H10	GCA_022344655.1	
	H1	GCA_022344665.1	
	EGS_44-159	GCA_022344695.1	
	EGS_44-110	GCA_022344705.1	
	D4	GCA_022344745.1	
	D3	GCA_022344765.1	
	C6	GCA_022344775.1	
	C4	GCA_022344825.1	
	C5	GCA_022344835.1	
	C25	GCA_022344855.1	
	C24	GCA_022344875.1	
	C21	GCA_022344885.1	
	C22	GCA_022344895.1	
	C20	GCA_022344905.1	
	C19	GCA_022344925.1	
	C14	GCA_022344955.1	
	C11	GCA_022344985.1	
	BC2-RLR-17S	GCA_022344995.1	
	Z6	GCA_022345075.1	
	Z8	GCA_022345085.1	
	Z5	GCA_022345095.1	
	Z4	GCA_022345105.1	
	Z3	GCA_022345155.1	
	Z13	GCA_022345165.1	
	Z14	GCA_022345175.1	
	Z15	GCA_022345185.1	
	Z12	GCA_022345235.1	
	Z11	GCA_022345245.1	
	Z1	GCA_022345265.1	
	Z10	GCA_022345285.1	
	Y8	GCA_022345305.1	
	Y7	GCA_022345335.1	
	Z2	GCA_022355435.1	
	Altal1	GCA_023079285.1	Shandong Agricultural University

	DZ	GCA_029891365.1	Hunan Agricultural University
Alternaria arborescens	EGS 39-128	GCA_000256225.1	Department of Plant Pathology, The Ohio State University
	FERA 675	GCA_004154835.1	NIAB-East Malling Research
	RGR 97.0013	GCA_004155955.1	
	MOD1-FUNGI6	GCA_004634205.1	USFDA
	NRRL 20593	GCA_013282825.1	US Department of Agriculture
	DET2008	GCA_020736785.1	Agriculture and Agri-Food Canada
	DET2035	GCA_040783815.1	
Alternaria arbusti	BMP 1465	GCA_024043155.1	Cornell University
Alternaria atra	MOD1-FUNGI7	GCA_004634305.1	USFDA
	CS162	GCA_907166805.1	Technical University Munich
Alternaria brassicae	J3	GCA_004936725.1	University of Delhi South Campus
	LPND	GCA_030704925.1	ICAR-Indian Agricultural Research Institute
Alternaria brassicicola	ATCC 96836	GCA_000174375.1	Washington University Genome Center
	Abra43	GCA_002796735.1	IRHS
	Altbr1	GCA_023079265.1	Shandong Agricultural University
	ATCC 96836	GCA_027569035.1	Northwestern University
	W 1	GCA_027569655.1	
Alternaria burnsii	CBS107.38	GCA_013036055.1	Northwest A&F University
	HNLF-44	GCA_021613345.1	Guangxi Institute of Botany
	/	GCA_951284595.1	Indian Institute of Technology
Alternaria conjuncta	BMP 0040	GCA_024043145.1	Cornell University
Alternaria consortialis	JCM 1940	GCA_001950455.1	RIKEN Center for Life Science Technologies
Alternaria dauci	CALB1	GCA_025504955.1	CAAS
Alternaria destruens	LH_A593	GCA_037044395.1	Qingdao Agricultural University
Alternaria ethzedia	BMP 0044	GCA_023757985.1	Cornell University
Alternaria gaisen	FERA 650	GCA_004156025.2	NIAB-East Malling Research
	X5	GCA_022345345.1	Beijing Forestry University
	X3	GCA_022345375.1	
	X2	GCA_022345385.1	
	X14	GCA_022345405.1	
	X13	GCA_022345445.1	
	X10	GCA_022345455.1	
	H9	GCA_022345465.1	
	H8	GCA_022345485.1	
	H7	GCA_022345505.1	
	H16	GCA_022345525.1	
	H14	GCA_022345565.1	

	D9	GCA_022345575.1	
	D8	GCA_022345585.1	
	D5	GCA_022345615.1	
	D7	GCA_022345635.1	
	D11	GCA_022345655.1	
	D10	GCA_022345675.1	
Alternaria gansuensis	LYZ1412	GCA_008973795.1<br>GCA_009289805.1	Lanzhou University
Alternaria hordeiaustralica	BMP 2776	GCA_023758085.1	Cornell University
Alternaria incomplexa	BMP 0042	GCA_024043165.1	Cornell University
Alternaria infectoria	BMP 0036	GCA_024043175.1	Cornell University
Alternaria longipes	CBS 540.94	GCA_019059555.1	Northwest A&F University
Alternaria metachromatica	BMP 0045	GCA_023757995.1	Cornell University
Alternaria novae-zelandiae	BMP 2774	GCA_023758075.1	Cornell University
Alternaria panax	BNCC115425	GCA_019702505.1	Anhui Agricultural University
Alternaria porri	Apn-Nashik	GCA_029783965.1	Rama Devi Women's University
Alternaria postmessia	BMP 2775	GCA_024291825.1	Cornell University
Alternaria rosae	MPI-PUGE-AT-0040	GCA_020736505.1	DOE Joint Genome Institute
	BMP 2777	GCA_024043135.1	Cornell University
Alternaria solani	HWC-168-2012p	GCA_002837235.1	Institute Of Microbiology Chinese Academy of Sciences
	NL03003	GCA_002952155.1	WUR
	TA-0410	GCA_036363465.1	Yunnan Nourmal University
Alternaria tenuissima	FERA 1082	GCA_004154745.1	NIAB-East Malling Research
Alternaria triticimaculans	BMP 0046	GCA_023758025.1	Cornell University
Alternaria triticina	CBS 763.84	GCA_022609215.1	Institute of Microbiology, Chinese Academy of Sciences
Alternaria ventricosa	BMP 2768	GCA_023758065.1	Cornell University
Alternaria viburni	BMP 2772	GCA_023758015.1	Cornell University

Table S2. Reference-Specific Target Sequences and Annotations for each *Alternaria* species.

Species	Reference-specific target sequence	Chromosomal location	Annotation	Sequence range
A. alstroemeriae	TTTGTCTATTCGTGCGGATGTACTA	no assembled	/	34-58
A. arborescens	TTTGTGAGGGAGGAGGCTATCGCAC	no assembled	hypothetical protein	173521-173545
A. brassicae	TTTATAATGCCATAACGGCCCAAGT	chromosome 8	/	1173812-1173836

A. gaisen	TTTCGAACAACGTCATCGAGATTGC	no assembled	/	695411-695435
A. triticimaculans	TTTACTGTGGGTTCTGAGATGCGTG	no assembled	/	34326-34350
A. atra	TTTGGCGATATCTAGGAATTGATGC	no assembled	uncharacterized protein	75848-75872
A. brassicicola	TTTCTGGACGCCTACAGCGTAGTCA	no assembled	/	568001-568025
A. burnsii	TTTGGCAGTGTGTGGGACACTTACG	no assembled	/	905214-905238
A. conjuncta	TTTGTGCGTTCACTGAAGAGTCTGG	no assembled	uncharacterized protein	1923108-1923132
A. consortialis	TTTGCTACGAACACAATGGATCACG	no assembled	/	89974-89998
A. dauci	TTTCTTAGCATTCCGCTCAGATCGT	no assembled	/	698600-698624
A. destruens	TTTGGGTGCATAGTTTGGTTACCAA	no assembled	/	113584-113608
A. ethzedia	TTTGTGTGCCTTGCTTCGCGACTCG	no assembled	/	39233-39257
A. gansuensis	TTTAACTCTTGCACTACAGGCTATA	no assembled	/	149613-149637
A. hordeiaustralica	TTTCTGCATATATGCGTAGCAGTGT	no assembled	uncharacterized protein	302193-302217
A. incomplexa	TTTCGGACCTTAGCATGTCCCGCAA	no assembled	uncharacterized protein	344924-344948
A. metachromatica	TTTGCGAACCTCTGGCAGTCATGAT	no assembled	uncharacterized protein	1442293-1442317
A. novae-zelandiae	TTTCCGATACAAGTTGCATTCTGCC	no assembled	uncharacterized protein	245541-245565
A. panax	TTTCTATGGCCGAGATTGCTGTCGC	no assembled	hypothetical protein	1535147-1535171
A. porri	TTTGCTGCTTCCATGCCTTATACGC	no assembled	/	1895100-1895124
A. postmessia	TTTGTCTCAAGGTACATGGACGCGA	no assembled	uncharacterized protein	183665-183689
A. rosae	TTTCATTACCGCGATGACGCAGTTG	no assembled	/	1595053-1595077
A. ventricosa	TTTATAGCGTACAACCGAAGACTGG	no assembled	Mannitol-1-phosphate 5-dehydrogenase	80045-80069
A. viburni	TTTCCCTACAGTCGGAGAATTAGTG	no assembled	hypothetical protein	2381784-2381808

Table S3. Primers for the seven species used in experimental validation.

Species	Primer(5'→3')
A. arbusti	F: GCTTCGTGATCCACTGATGGGTGAT<br>R: CAGTTTGCGAAGGAGCGAAGAACGT
A. infectoria	F: GCCTATCTGCTCGCCAAGATGTCTG<br>R: GCGTCCAATCAGCTTGGATGTAGTC
A. solani	F: AAACAGCAGATTTCTGCCCAGGTTT

	R: TCACCGCCGGTACATAGTTGATAGG
A. tenuissima	F: AATTCTTGGCTGTGTAGACATACGA<br>R: AGAGAGATACCCTCATCTGGAATGG
A. triticina	F: ATCTGGCTATTGGCTTGCATGATAT<br>R: TTAGTGTGTAAGGACAACCAGTTTG
A. alternata	F: ATGGCGGGAATAAGAAAGAGGAAAC<br>R: ATTAACGACACCTTGATTGGCTTCA
A.longipes	F: GGCAGTTGCATTGCGTTCATCTGAT<br>R: GTCGCGTCACACGAAGTATATCTCT

Table S4. crRNA for the seven species used in experimental validation.

Species	crRNA
A. arbusti	UAAUUUCUACUAAGUGUAGAUACCGACACCGCGUACUCUGUC
A. infectoria	UAAUUUCUACUAAGUGUAGAUCGCUCUAGUUGAUCCUCCACU
A. solani	UAAUUUCUACUAAGUGUAGAUUAGCGCCCCGAAGCAGAAUGG
A. tenuissima	UAAUUUCUACUAAGUGUAGAUUACUAUAUAUAUAUAACGCUU
A. triticina	UAAUUUCUACUAAGUGUAGAUGCGACGCGGAUCUAAUUGCAC
A. alternata	UAAUUUCUACUAAGUGUAGAUUAGAACACUCGACCUUGUCUG
A.longipes	UAAUUUCUACUAAGUGUAGAUCUUGGCGUAGCAUAGGAGCAC

A. arbusti

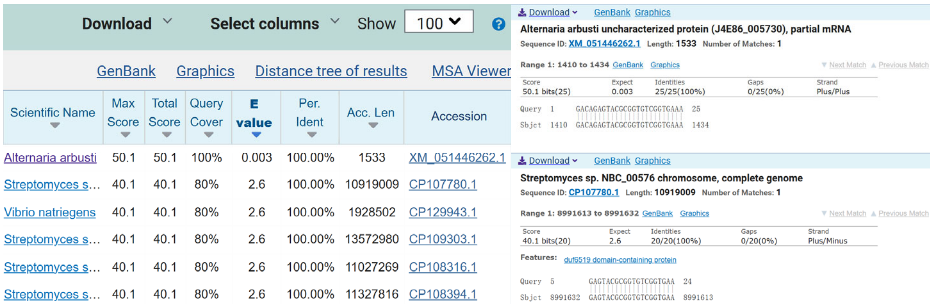
Download Select columns Show 100

GenBank Graphics Distance tree of results MSA Viewer

Scientific Name	Max Score	Total Score	Query Cover	E value	Per. Ident	Acc. Len	Accession
Alternaria arbusti	50.1	50.1	100%	0.003	100.00%	1533	XM_051446262.1
Streptomyces s...	40.1	40.1	80%	2.6	100.00%	10919009	CP107780.1
Vibrio natriegens	40.1	40.1	80%	2.6	100.00%	1928502	CP129943.1
Streptomyces s...	40.1	40.1	80%	2.6	100.00%	13572980	CP109303.1
Streptomyces s...	40.1	40.1	80%	2.6	100.00%	11027269	CP108316.1
Streptomyces s...	40.1	40.1	80%	2.6	100.00%	11327816	CP108394.1

Download GenBank Graphics
Alternaria arbusti uncharacterized protein (J4E86_005730), partial mRNA
Sequence ID: XM_051446262.1 Length: 1533 Number of Matches: 1
Range 1: 1410 to 1434 GenBank Graphics Next Match Previous Match

Score	Expect	Identities	Gaps	Strand
50.1 bits(25)	0.003	25/25(100%)	0/25(0%)	Plus/Plus

```
Query  1     GACAGAGTACGCGGTGTCGGTGAAA  25
Sbjct  1410  GACAGAGTACGCGGTGTCGGTGAAA  1434
```

Download GenBank Graphics
Streptomyces sp. NBC_00576 chromosome, complete genome
Sequence ID: CP107780.1 Length: 10919009 Number of Matches: 1
Range 1: 8991613 to 8991632 GenBank Graphics Next Match Previous Match

Score	Expect	Identities	Gaps	Strand
40.1 bits(20)	2.6	20/20(100%)	0/20(0%)	Plus/Minus

Features: duf6519 domain-containing protein

```
Query  5        GAGTACGCGGTGTCGGTGAA  24
Sbjct  8991632  GAGTACGCGGTGTCGGTGAA  8991613
```

A. infectoria

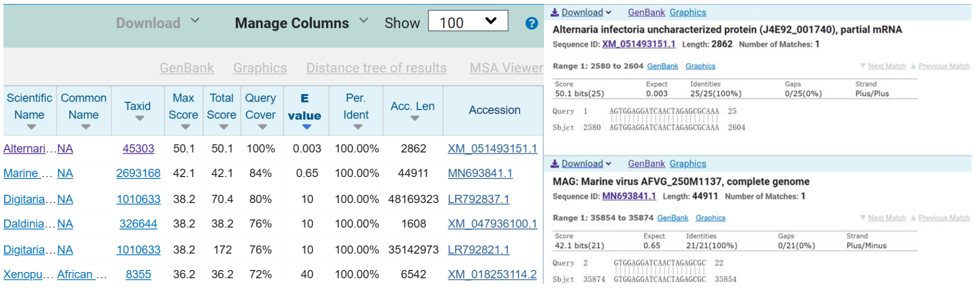
Download Manage Columns Show 100

GenBank Graphics Distance tree of results MSA Viewer

Scientific Name	Common Name	Taxid	Max Score	Total Score	Query Cover	E value	Per. Ident	Acc. Len	Accession
Alternari...	NA	45303	50.1	50.1	100%	0.003	100.00%	2862	XM_051493151.1
Marine ...	NA	2693168	42.1	42.1	84%	0.65	100.00%	44911	MN693841.1
Digitaria...	NA	1010633	38.2	70.4	80%	10	100.00%	48169323	LR792837.1
Daldinia...	NA	326644	38.2	38.2	76%	10	100.00%	1608	XM_047936100.1
Digitaria...	NA	1010633	38.2	172	76%	10	100.00%	35142973	LR792821.1
Xenopu...	African ...	8355	36.2	36.2	72%	40	100.00%	6542	XM_018253114.2

Download GenBank Graphics
Alternaria infectoria uncharacterized protein (J4E92_001740), partial mRNA
Sequence ID: XM_051493151.1 Length: 2862 Number of Matches: 1
Range 1: 2580 to 2604 GenBank Graphics Next Match Previous Match

Score	Expect	Identities	Gaps	Strand
50.1 bits(25)	0.003	25/25(100%)	0/25(0%)	Plus/Plus

```
Query  1     AGTGGAGGATCAACTAGAGCGCAAA  25
Sbjct  2580  AGTGGAGGATCAACTAGAGCGCAAA  2604
```

Download GenBank Graphics
MAG: Marine virus AFVG_250M1137, complete genome
Sequence ID: MN693841.1 Length: 44911 Number of Matches: 1
Range 1: 35854 to 35874 GenBank Graphics Next Match Previous Match

Score	Expect	Identities	Gaps	Strand
42.1 bits(21)	0.65	21/21(100%)	0/21(0%)	Plus/Minus

```
Query  2      GTGGAGGATCAACTAGAGCGC  22
Sbjct  35874  GTGGAGGATCAACTAGAGCGC  35854
```

A. solani

Download ⌄ Select columns ⌄ Show 100

GenBank Graphics Distance tree of results MSA Viewer

Scientific Name	Max Score	Total Score	Query Cover	E value	Per. Ident	Acc. Len	Accession
Bradyrhizobium…	38.2	38.2	76%	10	100.00%	9940904	CP096255.1
Aythya fuligula	38.2	38.2	76%	10	100.00%	663	XM_032200008.1
Marisediminicol…	38.2	38.2	76%	10	100.00%	3352609	CP017146.1
Meleagris gallo…	38.2	38.2	76%	10	100.00%	1276	XM_010720969.3
Anas platyrhync…	38.2	38.2	76%	10	100.00%	1264	XM_038188113.1
Anas platyrhync…	38.2	38.2	76%	10	100.00%	1141	XM_027471022.2

Download ⌄ GenBank Graphics

Bradyrhizobium barranii subsp. apii strain 1S5 chromosome, complete genome

Sequence ID: CP096255.1 Length: 9940904 Number of Matches: 1

Range 1: 9209992 to 9210010 GenBank Graphics ▼ Next Match ▲ Previous Match

Score	Expect	Identities	Gaps	Strand
38.2 bits(19)	10	19/19(100%)	0/19(0%)	Plus/Minus

```
Query  1        CCATTCTGCTTCGGGGCGC  19
                |||||||||||||||||||
Sbjct  9210010  CCATTCTGCTTCGGGGCGC  9209992
```

Download ⌄ GenBank Graphics

PREDICTED: Aythya fuligula myomaker, myoblast fusion factor (MYMK), mRNA

Sequence ID: XM_032200008.1 Length: 663 Number of Matches: 1

Range 1: 472 to 490 GenBank Graphics ▼ Next Match ▲ Previous Match

Score	Expect	Identities	Gaps	Strand
38.2 bits(19)	10	19/19(100%)	0/19(0%)	Plus/Plus

```
Query  4    TTCTGCTTCGGGGCGCTAG  22
            |||||||||||||||||||
Sbjct  472  TTCTGCTTCGGGGCGCTAG  490
```

A. tenuissima

Download ⌄ Select columns ⌄ Show 100

GenBank Graphics Distance tree of results MSA Viewer

Scientific Name	Max Score	Total Score	Query Cover	E value	Per. Ident	Acc. Len	Accession
Ctenopharyngo…	42.1	42.1	84%	0.68	100.00%	1766	XM_051908139.1
Tamlana crocina	42.1	42.1	84%	0.68	100.00%	3752971	CP158972.1
Arabidopsis tha…	42.1	402	96%	0.68	100.00%	29598828	LR797787.1
Lycium ferocissi…	40.1	40.1	80%	2.7	100.00%	366	XR_009407966.1
Danio rerio	40.1	40.1	80%	2.7	100.00%	114856	CR855323.18
Entelurus aequ…	40.1	40.1	80%	2.7	100.00%	3680	XM_062033927.1

Download ⌄ GenBank Graphics

PREDICTED: Ctenopharyngodon idella tubulin, alpha 7 like (tuba7l), mRNA

Sequence ID: XM_051908139.1 Length: 1766 Number of Matches: 1

Range 1: 277 to 297 GenBank Graphics ▼ Next Match ▲ Previous Match

Score	Expect	Identities	Gaps	Strand
42.1 bits(21)	0.68	21/21(100%)	0/21(0%)	Plus/Plus

```
Query  1    TTTCTACTATATATATATAAC  21
            |||||||||||||||||||||
Sbjct  277  TTTCTACTATATATATATAAC  297
```

Download ⌄ GenBank Graphics Sort by: E value

Arabidopsis thaliana genome assembly, chromosome: 1

Sequence ID: LR797787.1 Length: 29598828 Number of Matches: 12

Range 1: 8889738 to 8889758 GenBank Graphics ▼ Next Match ▲ Previous Match

Score	Expect	Identities	Gaps	Strand
42.1 bits(21)	0.68	21/21(100%)	0/21(0%)	Plus/Minus

```
Query  1        TTTCTACTATATATATATAAC  21
                |||||||||||||||||||||
Sbjct  8889758  TTTCTACTATATATATATAAC  8889738
```

A. triticina

Download ⌄ Select columns ⌄ Show 100

GenBank Graphics Distance tree of results MSA Viewer

Scientific Name	Max Score	Total Score	Query Cover	E value	Per. Ident	Acc. Len	Accession
Ixodes scapularis	38.2	38.2	76%	10	100.00%	112417	AC205634.1
Ixodes scapularis	38.2	38.2	76%	10	100.00%	114320	AC192419.4
Varroa jacobsoni	36.2	36.2	72%	40	100.00%	4434	XM_022845396.1
Varroa destructor	36.2	36.2	72%	40	100.00%	4938	XM_022801735.1
Varroa jacobsoni	36.2	36.2	72%	40	100.00%	4401	XM_022845397.1
Pseudocercosp…	36.2	36.2	72%	40	100.00%	1545	XM_007928371.1

Download ⌄ GenBank Graphics

Ixodes scapularis BAC ISG1-06P02, complete sequence

Sequence ID: AC205634.1 Length: 112417 Number of Matches: 1

Range 1: 52282 to 52300 GenBank Graphics ▼ Next Match ▲ Previous Match

Score	Expect	Identities	Gaps	Strand
38.2 bits(19)	10	19/19(100%)	0/19(0%)	Plus/Minus

```
Query  7      TTAGATCCGCGTCGCGAAA  25
              |||||||||||||||||||
Sbjct  52300  TTAGATCCGCGTCGCGAAA  52282
```

Download ⌄ GenBank Graphics

Ixodes scapularis, clone XX-43A1, complete sequence

Sequence ID: AC192419.4 Length: 114320 Number of Matches: 1

Range 1: 81047 to 81065 GenBank Graphics ▼ Next Match ▲ Previous Match

Score	Expect	Identities	Gaps	Strand
38.2 bits(19)	10	19/19(100%)	0/19(0%)	Plus/Minus

```
Query  7      TTAGATCCGCGTCGCGAAA  25
              |||||||||||||||||||
Sbjct  81065  TTAGATCCGCGTCGCGAAA  81047
```

A. alternata

Download ⌄ Select columns ⌄ Show 100

GenBank Graphics Distance tree of results MSA Viewer

Scientific Name	Max Score	Total Score	Query Cover	E value	Per. Ident	Acc. Len	Accession
Alternaria altern…	50.1	50.1	100%	0.003	100.00%	1809	XM_018523813.1
Fusarium subgl…	38.2	38.2	76%	10	100.00%	1608	XM_036687090.1
Exophiala bona…	36.2	36.2	72%	40	100.00%	2016	XM_064851981.1
Simochromis di…	36.2	36.2	72%	40	100.00%	3191	XM_040018733.1
Furfurilactobacil…	36.2	36.2	72%	40	100.00%	2804324	CP040576.1
Simochromis di…	36.2	36.2	72%	40	100.00%	2544	XM_040018732.1

Download ⌄ GenBank Graphics

Alternaria alternata alpha/beta-hydrolase partial mRNA

Sequence ID: XM_018523813.1 Length: 1809 Number of Matches: 1

Range 1: 887 to 911 GenBank Graphics ▼ Next Match ▲ Previous Match

Score	Expect	Identities	Gaps	Strand
50.1 bits(25)	0.003	25/25(100%)	0/25(0%)	Plus/Plus

```
Query  1    CAGACAAGGTCGAGTGTTCTACAAA  25
            |||||||||||||||||||||||||
Sbjct  887  CAGACAAGGTCGAGTGTTCTACAAA  911
```

Download ⌄ GenBank Graphics

Fusarium subglutinans uncharacterized protein (FSUBG_8660), partial mRNA

Sequence ID: XM_036687090.1 Length: 1608 Number of Matches: 1

Range 1: 760 to 778 GenBank Graphics ▼ Next Match ▲ Previous Match

Score	Expect	Identities	Gaps	Strand
38.2 bits(19)	10	19/19(100%)	0/19(0%)	Plus/Plus

```
Query  3    GACAAGGTCGAGTGTTCTA  21
            |||||||||||||||||||
Sbjct  760  GACAAGGTCGAGTGTTCTA  778
```

A. longipes

Download Select columns Show 100

GenBank Graphics Distance tree of results MSA Viewer

Scientific Name	Max Score	Total Score	Query Cover	E value	Per. Ident	Acc. Len	Accession
Gopherus flavo…	38.2	38.2	76%	10	100.00%	912	XM_050944396.1
Timema tahoe	38.2	38.2	76%	10	100.00%	25694	OE008173.1
Gopherus flavo…	38.2	38.2	76%	10	100.00%	1710	XM_050944392.1
Gopherus flavo…	38.2	38.2	76%	10	100.00%	3469	XM_050944391.1
Ochotona curzo…	36.2	36.2	72%	40	100.00%	1819	XM_040967513.1
Dromiciops glir…	36.2	36.2	72%	40	100.00%	1933	XM_043968176.1

Download GenBank Graphics

PREDICTED: Gopherus flavomarginatus myosin light chain 5 (MYL5), transcript variant X7,

Sequence ID: XM_050944396.1 Length: 912 Number of Matches: 1

Range 1: 18 to 36 GenBank Graphics ▼ Next Match ▲ Previous Match

Score	Expect	Identities	Gaps	Strand
38.2 bits(19)	10	19/19(100%)	0/19(0%)	Plus/Minus

```
Query  6    CCTATGCTACGCCAAGGAA  24
            |||||||||||||||||||
Sbjct  36   CCTATGCTACGCCAAGGAA  18
```

Download GenBank Graphics

4_Tte_b3v08

Sequence ID: OE008173.1 Length: 25694 Number of Matches: 1

Range 1: 3876 to 3894 GenBank Graphics ▼ Next Match ▲ Previous Match

Score	Expect	Identities	Gaps	Strand
38.2 bits(19)	10	19/19(100%)	0/19(0%)	Plus/Minus

```
Query  5     TCCTATGCTACGCCAAGGA  23
             |||||||||||||||||||
Sbjct  3894  TCCTATGCTACGCCAAGGA  3876
```

A. alstroemeriae

Download Select columns Show 100

GenBank Graphics Distance tree of results MSA Viewer

Scientific Name	Max Score	Total Score	Query Cover	E value	Per. Ident	Acc. Len	Accession
Neobacillus sp. …	38.2	38.2	76%	11	100.00%	6085354	CP126107.1
Diachasma allo…	36.2	36.2	72%	42	100.00%	2994	XM_015254232.1
Diachasma allo…	36.2	36.2	72%	42	100.00%	3353	XM_015254223.1
Streptococcus s…	36.2	36.2	72%	42	100.00%	2551068	CP160400.1
Diachasma allo…	36.2	36.2	72%	42	100.00%	3607	XM_015254241.1
Colydium elong…	36.2	36.2	72%	42	100.00%	37133187	OZ210023.1

Download GenBank Graphics

Neobacillus sp. SuZ13 chromosome, complete genome

Sequence ID: CP126107.1 Length: 6085354 Number of Matches: 1

Range 1: 3220785 to 3220803 GenBank Graphics ▼ Next Match ▲ Previous Match

Score	Expect	Identities	Gaps	Strand
38.2 bits(19)	11	19/19(100%)	0/19(0%)	Plus/Minus

```
Query  7        ATCCGCACGAATAGACAAA  25
                |||||||||||||||||||
Sbjct  3220803  ATCCGCACGAATAGACAAA  3220785
```

Download GenBank Graphics

PREDICTED: Diachasma alloeum FYVE, RhoGEF and PH domain-containing protein 1 (LOC1

Sequence ID: XM_015254232.1 Length: 2994 Number of Matches: 1

Range 1: 768 to 785 GenBank Graphics ▼ Next Match ▲ Previous Match

Score	Expect	Identities	Gaps	Strand
36.2 bits(18)	42	18/18(100%)	0/18(0%)	Plus/Plus

```
Query  8    TCCGCACGAATAGACAAA  25
            ||||||||||||||||||
Sbjct  768  TCCGCACGAATAGACAAA  785
```

A. arborescens

Download Select columns Show 100

GenBank Graphics Distance tree of results MSA Viewer

Scientific Name	Max Score	Total Score	Query Cover	E value	Per. Ident	Acc. Len	Accession
Mesocricetus a…	38.2	38.2	76%	10	100.00%	8778	XM_013121179.3
Mesocricetus a…	38.2	38.2	76%	10	100.00%	8621	XM_021233520.2
Mesocricetus a…	38.2	38.2	76%	10	100.00%	8700	XM_021233522.2
Candidatus Zixi…	38.2	38.2	76%	10	100.00%	2846532	CP070796.1
Callorhinchus milii	38.2	38.2	76%	10	100.00%	2423	XM_007901758.2
Mesocricetus a…	38.2	38.2	76%	10	100.00%	9072	XM_040747636.1

Download GenBank Graphics

PREDICTED: Mesocricetus auratus thyroid hormone receptor beta (Thrb), transcript variant

Sequence ID: XM_013121179.3 Length: 8778 Number of Matches: 1

Range 1: 3666 to 3684 GenBank Graphics ▼ Next Match ▲ Previous Match

Score	Expect	Identities	Gaps	Strand
38.2 bits(19)	10	19/19(100%)	0/19(0%)	Plus/Minus

```
Query  1     TTTGTGAGGGAGGAGGCTA  19
             |||||||||||||||||||
Sbjct  3684  TTTGTGAGGGAGGAGGCTA  3666
```

Download GenBank Graphics

PREDICTED: Mesocricetus auratus thyroid hormone receptor beta (Thrb), transcript variant

Sequence ID: XM_021233520.2 Length: 8621 Number of Matches: 1

Range 1: 3509 to 3527 GenBank Graphics ▼ Next Match ▲ Previous Match

Score	Expect	Identities	Gaps	Strand
38.2 bits(19)	10	19/19(100%)	0/19(0%)	Plus/Minus

```
Query  1     TTTGTGAGGGAGGAGGCTA  19
             |||||||||||||||||||
Sbjct  3527  TTTGTGAGGGAGGAGGCTA  3509
```

A. brassicae

Download Select columns Show 100

GenBank Graphics Distance tree of results MSA Viewer

Scientific Name	Max Score	Total Score	Query Cover	E value	Per. Ident	Acc. Len	Accession
Sporomusa mal…	42.1	74.3	84%	0.65	100.00%	5312210	CP155572.1
Humulus lupulus	40.1	40.1	80%	2.6	100.00%	1933	XM_062242929.1
Komagataeibac…	40.1	40.1	80%	2.6	100.00%	221732	CP036405.1
Trueperella pyo…	36.2	36.2	72%	40	100.00%	2415007	CP050810.1
Marinobacter s…	36.2	36.2	72%	40	100.00%	4264018	CP045324.1
Marinobacter s…	36.2	36.2	72%	40	100.00%	4256935	CP045367.1

Download GenBank Graphics Sort by: E value

Sporomusa malonica strain DSM 5090 chromosome, complete genome

Sequence ID: CP155572.1 Length: 5312210 Number of Matches: 2

Range 1: 4036981 to 4037001 GenBank Graphics ▼ Next Match ▲ Previous Match

Score	Expect	Identities	Gaps	Strand
42.1 bits(21)	0.65	21/21(100%)	0/21(0%)	Plus/Minus

```
Query  5        GGGCCGTTATGGCATTATAAA  25
                |||||||||||||||||||||
Sbjct  4037001  GGGCCGTTATGGCATTATAAA  4036981
```

Range 2: 2066128 to 2066143 GenBank Graphics ▼ Next Match ▲ Previous Match ⇑ First Match

Score	Expect	Identities	Gaps	Strand
32.2 bits(16)	623	16/16(100%)	0/16(0%)	Plus/Plus

```
Query  10       GTTATGGCATTATAAA  25
                ||||||||||||||||
Sbjct  2066128  GTTATGGCATTATAAA  2066143
```

A. gaisen

Download Select columns Show 100

GenBank Graphics Distance tree of results MSA Viewer

Scientific Name	Max Score	Total Score	Query Cover	E value	Per. Ident	Acc. Len	Accession
Caulobacter se…	40.1	40.1	80%	2.6	100.00%	5173281	CP082923.1
Gasterosteus a…	38.2	38.2	76%	10	100.00%	5589	XM_040173589.1
Humisphaera b…	38.2	38.2	76%	10	100.00%	7189892	CP063458.1
Anarrhichthys o…	38.2	38.2	76%	10	100.00%	6198	XM_031865665.1
Bosea vestrisii	38.2	38.2	76%	10	100.00%	5934299	CP126307.1
Gasterosteus a…	38.2	38.2	76%	10	100.00%	5678	XM_040173583.1

Download GenBank Graphics

Caulobacter segnis strain CBR1 chromosome, complete genome
Sequence ID: CP082923.1 Length: 5173281 Number of Matches: 1

Range 1: 2694362 to 2694381 GenBank Graphics ▼ Next Match ▲ Previous Match

Score	Expect	Identities	Gaps	Strand
40.1 bits(20)	2.6	20/20(100%)	0/20(0%)	Plus/Plus

```
Query  6        CTCGATGACGTTGTTCGAAA  25
                ||||||||||||||||||||
Sbjct  2694362  CTCGATGACGTTGTTCGAAA  2694381
```

Download GenBank Graphics

PREDICTED: Gasterosteus aculeatus aculeatus anoctamin-4 (LOC120817397), transcript va
Sequence ID: XM_040173589.1 Length: 5589 Number of Matches: 1

Range 1: 4600 to 4618 GenBank Graphics ▼ Next Match ▲ Previous Match

Score	Expect	Identities	Gaps	Strand
38.2 bits(19)	10	19/19(100%)	0/19(0%)	Plus/Minus

```
Query  3     AATCTCGATGACGTTGTTC  21
             |||||||||||||||||||
Sbjct  4618  AATCTCGATGACGTTGTTC  4600
```

A. triticimaculans

Download Select columns Show 100

GenBank Graphics Distance tree of results MSA Viewer

Scientific Name	Max Score	Total Score	Query Cover	E value	Per. Ident	Acc. Len	Accession
Legionella israe…	38.2	38.2	76%	10	100.00%	2967881	CP038254.1
Legionella israe…	38.2	38.2	76%	10	100.00%	3046673	CP041668.1
Ilyonectria robu…	38.2	38.2	76%	10	100.00%	2956	XM_046255646.1
Oligella urethralis	38.2	38.2	76%	10	100.00%	2580519	CP137240.1
Meleagris gallo…	38.2	38.2	76%	10	100.00%	10809360	HG999699.4
Legionella israe…	38.2	38.2	76%	10	100.00%	3217863	CP038273.1

Download GenBank Graphics

Legionella israelensis strain HL-0427-4011 chromosome, complete genome
Sequence ID: CP038254.1 Length: 2967881 Number of Matches: 1

Range 1: 2680913 to 2680931 GenBank Graphics ▼ Next Match ▲ Previous Match

Score	Expect	Identities	Gaps	Strand
38.2 bits(19)	10	19/19(100%)	0/19(0%)	Plus/Plus

```
Query  7        TCTCAGAACCCACAGTAAA  25
                |||||||||||||||||||
Sbjct  2680913  TCTCAGAACCCACAGTAAA  2680931
```

Download GenBank Graphics

Legionella israelensis strain L18-01051 chromosome, complete genome
Sequence ID: CP041668.1 Length: 3046673 Number of Matches: 1

Range 1: 15808 to 15826 GenBank Graphics ▼ Next Match ▲ Previous Match

Score	Expect	Identities	Gaps	Strand
38.2 bits(19)	10	19/19(100%)	0/19(0%)	Plus/Plus

```
Query  7      TCTCAGAACCCACAGTAAA  25
              |||||||||||||||||||
Sbjct  15808  TCTCAGAACCCACAGTAAA  15826
```

A. atra

GenBank Graphics Distance tree of results MSA Viewer

Scientific Name	Max Score	Total Score	Query Cover	E value	Per. Ident	Acc. Len	Accession
Streptococcus …	38.2	38.2	76%	10	100.00%	2196662	CP000725.1
Streptococcus …	38.2	38.2	76%	10	100.00%	2204031	CP113953.1
Streptococcus …	38.2	38.2	76%	10	100.00%	2196660	LR134291.1
Streptococcus …	38.2	38.2	76%	10	100.00%	2185555	LR594049.1
Streptococcus …	38.2	38.2	76%	10	100.00%	2154796	LS483375.1
Streptococcus …	38.2	38.2	76%	10	100.00%	2196595	CP046328.1

Download GenBank Graphics

Streptococcus gordonii str. Challis substr. CH1, complete genome
Sequence ID: CP000725.1 Length: 2196662 Number of Matches: 1

Range 1: 2187743 to 2187761 GenBank Graphics ▼ Next Match ▲ Previous Match

Score	Expect	Identities	Gaps	Strand
38.2 bits(19)	10	19/19(100%)	0/19(0%)	Plus/Plus

```
Query  4        TCAATTCCTAGATATCGCC  22
                |||||||||||||||||||
Sbjct  2187743  TCAATTCCTAGATATCGCC  2187761
```

Download GenBank Graphics

Streptococcus gordonii strain CW chromosome
Sequence ID: CP113953.1 Length: 2204031 Number of Matches: 1

Range 1: 2194947 to 2194965 GenBank Graphics ▼ Next Match ▲ Previous Match

Score	Expect	Identities	Gaps	Strand
38.2 bits(19)	10	19/19(100%)	0/19(0%)	Plus/Plus

```
Query  4        TCAATTCCTAGATATCGCC  22
                |||||||||||||||||||
Sbjct  2194947  TCAATTCCTAGATATCGCC  2194965
```

A. brassicicola

GenBank Graphics Distance tree of results MSA Viewer

Scientific Name	Max Score	Total Score	Query Cover	E value	Per. Ident	Acc. Len	Accession
Halobacterium …	38.2	38.2	76%	10	100.00%	2178608	CP038631.1
Halobacterium …	38.2	38.2	76%	10	100.00%	2000962	AM774415.1
Halobacterium …	38.2	38.2	76%	10	100.00%	1969683	CP146625.1
Halobacterium …	38.2	38.2	76%	10	100.00%	1959163	CP128377.3
Halobacterium …	38.2	38.2	76%	10	100.00%	2014239	BK010829.1
Poecilia reticulata	38.2	38.2	76%	10	100.00%	1629	XM_008437911.2

Download GenBank Graphics

Halobacterium salinarum strain 91-R6 chromosome, complete genome
Sequence ID: CP038631.1 Length: 2178608 Number of Matches: 1

Range 1: 973052 to 973070 GenBank Graphics ▼ Next Match ▲ Previous Match

Score	Expect	Identities	Gaps	Strand
38.2 bits(19)	10	19/19(100%)	0/19(0%)	Plus/Plus

```
Query  7       CGCTGTAGGCGTCCAGAAA  25
               |||||||||||||||||||
Sbjct  973052  CGCTGTAGGCGTCCAGAAA  973070
```

Download GenBank Graphics

Halobacterium salinarum R1 complete genome
Sequence ID: AM774415.1 Length: 2000962 Number of Matches: 1

Range 1: 633712 to 633730 GenBank Graphics ▼ Next Match ▲ Previous Match

Score	Expect	Identities	Gaps	Strand
38.2 bits(19)	10	19/19(100%)	0/19(0%)	Plus/Plus

```
Query  7       CGCTGTAGGCGTCCAGAAA  25
               |||||||||||||||||||
Sbjct  633712  CGCTGTAGGCGTCCAGAAA  633730
```

A. burnsii

GenBank Graphics Distance tree of results MSA Viewer

Scientific Name	Max Score	Total Score	Query Cover	E value	Per. Ident	Acc. Len	Accession
Heterocephalus…	36.2	70.4	92%	40	95.45%	73868007	OZ001124.1
Heterocephalus…	36.2	70.4	92%	40	95.45%	71916000	OZ001081.1
Equus caballus	34.2	66.4	92%	156	100.00%	88986268	OZ014261.2
Brassica napus	36.2	36.2	88%	40	95.45%	44786494	CP123878.1
Amphiprion oce…	36.2	36.2	88%	40	95.45%	5310	XM_035954981.2
Amphiprion oce…	36.2	36.2	88%	40	95.45%	4897	XM_055015972.1

Download GenBank Graphics Sort by: E value

Heterocephalus glaber genome assembly, chromosome: 16
Sequence ID: OZ001124.1 Length: 73868007 Number of Matches: 2

Range 1: 3244688 to 3244709 GenBank Graphics ▼ Next Match ▲ Previous Match

Score	Expect	Identities	Gaps	Strand
36.2 bits(18)	40	21/22(95%)	0/22(0%)	Plus/Minus

```
Query  4        AAGTGTCCCACACACTGCCAAA  25
                |||||||||| |||||||||||
Sbjct  3244709  AAGTGTCCCAGACACTGCCAAA  3244688
```

Range 2: 38342191 to 38342207 GenBank Graphics ▼ Next Match ▲ Previous Match ▲ First Match

Score	Expect	Identities	Gaps	Strand
34.2 bits(17)	156	17/17(100%)	0/17(0%)	Plus/Plus

```
Query  3         TAAGTGTCCCACACACT  19
                 |||||||||||||||||
Sbjct  38342191  TAAGTGTCCCACACACT  38342207
```

A. conjuncta

GenBank Graphics Distance tree of results MSA Viewer

Scientific Name	Max Score	Total Score	Query Cover	E value	Per. Ident	Acc. Len	Accession
Alternaria conju...	50.1	50.1	100%	0.003	100.00%	2634	XM_051469304.1
Lactiplantibacill...	38.2	38.2	76%	10	100.00%	3255701	CP103911.1
Lactiplantibacill...	38.2	38.2	76%	10	100.00%	3255701	CP103910.1
Lactiplantibacill...	38.2	38.2	76%	10	100.00%	3217780	CP096862.1
Lactiplantibacill...	38.2	38.2	76%	10	100.00%	3163810	OZ061574.1
Lactiplantibacill...	38.2	38.2	76%	10	100.00%	3212731	CP134072.1

Download GenBank Graphics

Alternaria conjuncta endoribonuclease ysh1 (YSH1), partial mRNA

Sequence ID: XM_051469304.1 Length: 2634 Number of Matches: 1

Range 1: 1009 to 1033 GenBank Graphics ▼ Next Match ▲ Previous Match

Score	Expect	Identities	Gaps	Strand
50.1 bits(25)	0.003	25/25(100%)	0/25(0%)	Plus/Minus

```
Query  1     CCAGACTCTTCAGTGAACGCACAAA  25
             |||||||||||||||||||||||||
Sbjct  1033  CCAGACTCTTCAGTGAACGCACAAA  1009
```

Download GenBank Graphics

Lactiplantibacillus plantarum strain SRCM210580 chromosome, complete genome

Sequence ID: CP103911.1 Length: 3255701 Number of Matches: 1

Range 1: 1836885 to 1836903 GenBank Graphics ▼ Next Match ▲ Previous Match

Score	Expect	Identities	Gaps	Strand
38.2 bits(19)	10	19/19(100%)	0/19(0%)	Plus/Plus

```
Query  7        TCTTCAGTGAACGCACAAA  25
                |||||||||||||||||||
Sbjct  1836885  TCTTCAGTGAACGCACAAA  1836903
```

A. consortialis

GenBank Graphics Distance tree of results MSA Viewer

Scientific Name	Max Score	Total Score	Query Cover	E value	Per. Ident	Acc. Len	Accession
Elephas maxim...	38.2	38.2	76%	10	100.00%	5264	XM_049862191.1
Loxodonta afric...	38.2	38.2	76%	10	100.00%	4612	XM_003409883.4
Sardina pilchar...	38.2	38.2	76%	10	100.00%	6795	XM_062533628.1
Mus musculus	36.2	36.2	72%	40	100.00%	219370	AC123935.4
Mus musculus	36.2	36.2	72%	40	100.00%	139149	AC115832.14
Oryza sativa Ja...	36.2	36.2	72%	40	100.00%	172238	AC145381.4

Download GenBank Graphics

PREDICTED: Elephas maximus indicus protein kinase cAMP-dependent type II regulatory su

Sequence ID: XM_049862191.1 Length: 5264 Number of Matches: 1

Range 1: 3159 to 3177 GenBank Graphics ▼ Next Match ▲ Previous Match

Score	Expect	Identities	Gaps	Strand
38.2 bits(19)	10	19/19(100%)	0/19(0%)	Plus/Minus

```
Query  7     CCATTGTGTTCGTAGCAAA  25
             |||||||||||||||||||
Sbjct  3177  CCATTGTGTTCGTAGCAAA  3159
```

Download GenBank Graphics

PREDICTED: Loxodonta africana protein kinase cAMP-dependent type II regulatory subunit

Sequence ID: XM_003409883.4 Length: 4612 Number of Matches: 1

Range 1: 2946 to 2964 GenBank Graphics ▼ Next Match ▲ Previous Match

Score	Expect	Identities	Gaps	Strand
38.2 bits(19)	10	19/19(100%)	0/19(0%)	Plus/Minus

```
Query  7     CCATTGTGTTCGTAGCAAA  25
             |||||||||||||||||||
Sbjct  2964  CCATTGTGTTCGTAGCAAA  2946
```

A. dauci

GenBank Graphics Distance tree of results MSA Viewer

Scientific Name	Max Score	Total Score	Query Cover	E value	Per. Ident	Acc. Len	Accession
Gallus gallus	34.2	34.2	84%	156	95.24%	4929	XM_046916278.1
Gallus gallus	34.2	34.2	84%	156	95.24%	6752	XM_046916277.1
Gallus gallus	34.2	34.2	84%	156	95.24%	10040	XM_046916276.1
Coprococcus c...	34.2	34.2	84%	156	95.24%	3373067	CP070062.1
Coprococcus c...	34.2	34.2	84%	156	95.24%	3373066	CP102277.1
Bacidia gigante...	34.2	34.2	84%	156	95.24%	1200	XM_044813363.1

Download GenBank Graphics

PREDICTED: Gallus gallus ligand dependent nuclear receptor corepressor like (LCORL), tran

Sequence ID: XM_046916278.1 Length: 4929 Number of Matches: 1

Range 1: 2782 to 2802 GenBank Graphics ▼ Next Match ▲ Previous Match

Score	Expect	Identities	Gaps	Strand
34.2 bits(17)	156	20/21(95%)	0/21(0%)	Plus/Plus

```
Query  5     TCTGAGCGGAATGCTAAGAAA  25
             |||||| ||||||||||||||
Sbjct  2782  TCTGAGAGGAATGCTAAGAAA  2802
```

Download GenBank Graphics

PREDICTED: Gallus gallus ligand dependent nuclear receptor corepressor like (LCORL), tran

Sequence ID: XM_046916277.1 Length: 6752 Number of Matches: 1

Range 1: 1950 to 1970 GenBank Graphics ▼ Next Match ▲ Previous Match

Score	Expect	Identities	Gaps	Strand
34.2 bits(17)	156	20/21(95%)	0/21(0%)	Plus/Plus

```
Query  5     TCTGAGCGGAATGCTAAGAAA  25
             |||||| ||||||||||||||
Sbjct  1950  TCTGAGAGGAATGCTAAGAAA  1970
```

A. destruens

GenBank Graphics Distance tree of results MSA Viewer

Scientific Name	Max Score	Total Score	Query Cover	E value	Per. Ident	Acc. Len	Accession
Oryza sativa in...	40.1	40.1	80%	2.5	100.00%	28602018	AP027392.1
Oryza sativa in...	40.1	40.1	80%	2.5	100.00%	32205552	CP141115.1
Oryza sativa In...	40.1	40.1	80%	2.5	100.00%	30492302	CP054683.1
Oryza sativa Ja...	40.1	40.1	80%	2.5	100.00%	149198	AP004751.3
Morone saxatilis	38.2	38.2	76%	10	100.00%	2042	XM_035660745.1
Oryza brachyan...	38.2	38.2	76%	10	100.00%	3202	XM_006651697.3

Download GenBank Graphics

Oryza sativa indica subgroup Inn Ma Yebaw DNA, chromosome: 8

Sequence ID: AP027392.1 Length: 28602018 Number of Matches: 1

Range 1: 7634809 to 7634828 GenBank Graphics ▼ Next Match ▲ Previous Match

Score	Expect	Identities	Gaps	Strand
40.1 bits(20)	2.5	20/20(100%)	0/20(0%)	Plus/Minus

```
Query  6        AACCAAACTATGCACCCAAA  25
                ||||||||||||||||||||
Sbjct  7634828  AACCAAACTATGCACCCAAA  7634809
```

Download GenBank Graphics

Oryza sativa indica subgroup cultivar ZH8015 chromosome 08

Sequence ID: CP141115.1 Length: 32205552 Number of Matches: 1

Range 1: 8203282 to 8203301 GenBank Graphics ▼ Next Match ▲ Previous Match

Score	Expect	Identities	Gaps	Strand
40.1 bits(20)	2.5	20/20(100%)	0/20(0%)	Plus/Minus

```
Query  6        AACCAAACTATGCACCCAAA  25
                ||||||||||||||||||||
Sbjct  8203301  AACCAAACTATGCACCCAAA  8203282
```

A. ethzedia

GenBank Graphics Distance tree of results MSA Viewer

Scientific Name	Max Score	Total Score	Query Cover	E value	Per. Ident	Acc. Len	Accession
Streptomyces t...	38.2	38.2	76%	10	100.00%	8628007	CP108737.1
Streptomyces t...	38.2	38.2	76%	10	100.00%	8552987	CP108419.1
Streptomyces t...	38.2	38.2	76%	10	100.00%	8613235	CP107952.1
Timema bartmani	38.2	38.2	76%	10	100.00%	12719	OD573762.1
Streptomyces t...	38.2	38.2	76%	10	100.00%	8538055	CP107742.1
Streptomyces t...	38.2	38.2	76%	10	100.00%	8649157	CP107884.1

Download GenBank Graphics

Streptomyces tubercidicus strain NBC_00975 chromosome, complete genome

Sequence ID: CP108737.1 Length: 8628007 Number of Matches: 1

Range 1: 309520 to 309538 GenBank Graphics ▼ Next Match ▲ Previous Match

Score	Expect	Identities	Gaps	Strand
38.2 bits(19)	10	19/19(100%)	0/19(0%)	Plus/Plus

```
Query  7       GCGAAGCAAGGCACACAAA  25
               |||||||||||||||||||
Sbjct  309520  GCGAAGCAAGGCACACAAA  309538
```

Download GenBank Graphics

Streptomyces tubercidicus strain NBC_01297 chromosome, complete genome

Sequence ID: CP108419.1 Length: 8552987 Number of Matches: 1

Range 1: 8227919 to 8227937 GenBank Graphics ▼ Next Match ▲ Previous Match

Score	Expect	Identities	Gaps	Strand
38.2 bits(19)	10	19/19(100%)	0/19(0%)	Plus/Minus

```
Query  7        GCGAAGCAAGGCACACAAA  25
                |||||||||||||||||||
Sbjct  8227937  GCGAAGCAAGGCACACAAA  8227919
```

A. gansuensis

GenBank Graphics Distance tree of results MSA Viewer

Scientific Name	Max Score	Total Score	Query Cover	E value	Per. Ident	Acc. Len	Accession
Diceros bicornis…	38.2	38.2	76%	10	100.00%	13940	XM_058536662.1
Diceros bicornis…	38.2	38.2	76%	10	100.00%	13967	XM_058536666.1
Diceros bicornis…	38.2	38.2	76%	10	100.00%	13925	XM_058536671.1
Diceros bicornis…	38.2	38.2	76%	10	100.00%	11718	XM_058536674.1
Diceros bicornis…	38.2	38.2	76%	10	100.00%	13928	XM_058536670.1
Diceros bicornis…	38.2	38.2	76%	10	100.00%	14224	XM_058536661.1

Download GenBank Graphics

PREDICTED: Diceros bicornis minor teneurin transmembrane protein 1 (TENM1), transcript

Sequence ID: XM_058536662.1 Length: 13940 Number of Matches: 1

Range 1: 12284 to 12302 GenBank Graphics ▼ Next Match ▲ Previous Match

Score	Expect	Identities	Gaps	Strand
38.2 bits(19)	10	19/19(100%)	0/19(0%)	Plus/Minus

```
Query  7      CTGTAGTGCAAGAGTTAAA  25
              |||||||||||||||||||
Sbjct  12302  CTGTAGTGCAAGAGTTAAA  12284
```

Download GenBank Graphics

PREDICTED: Diceros bicornis minor teneurin transmembrane protein 1 (TENM1), transcript

Sequence ID: XM_058536666.1 Length: 13967 Number of Matches: 1

Range 1: 12311 to 12329 GenBank Graphics ▼ Next Match ▲ Previous Match

Score	Expect	Identities	Gaps	Strand
38.2 bits(19)	10	19/19(100%)	0/19(0%)	Plus/Minus

```
Query  7      CTGTAGTGCAAGAGTTAAA  25
              |||||||||||||||||||
Sbjct  12329  CTGTAGTGCAAGAGTTAAA  12311
```

A. hordeiaustralica

GenBank Graphics Distance tree of results MSA Viewer

Scientific Name	Max Score	Total Score	Query Cover	E value	Per. Ident	Acc. Len	Accession
Ictalurus puncta…	34.2	34.2	84%	156	95.24%	2628	XM_053680790.1
Ictalurus puncta…	34.2	34.2	84%	156	95.24%	2648	XM_017484473.3
Ictalurus furcatus	34.2	34.2	84%	156	95.24%	2802	XM_053625484.1
Paenibacillus m…	38.2	38.2	76%	10	100.00%	6983959	CP118270.1
Dromaius nova…	38.2	38.2	76%	10	100.00%	8355	XM_064504035.1
Paludibacterac…	38.2	38.2	76%	10	100.00%	2518691	OY760143.1

Download GenBank Graphics

PREDICTED: Ictalurus punctatus prolyl 3-hydroxylase 3 (p3h3), transcript variant X1, mRNA

Sequence ID: XM_053680790.1 Length: 2628 Number of Matches: 1

Range 1: 2514 to 2534 GenBank Graphics ▼ Next Match ▲ Previous Match

Score	Expect	Identities	Gaps	Strand
34.2 bits(17)	156	20/21(95%)	0/21(0%)	Plus/Minus

```
Query  5     TGCTACGCATATATGCAGAAA  25
             |||||||||||||| ||||||
Sbjct  2534  TGCTACGCATATATACAGAAA  2514
```

Download GenBank Graphics

PREDICTED: Ictalurus punctatus prolyl 3-hydroxylase 3 (p3h3), transcript variant X2, mRNA

Sequence ID: XM_017484473.3 Length: 2648 Number of Matches: 1

Range 1: 2534 to 2554 GenBank Graphics ▼ Next Match ▲ Previous Match

Score	Expect	Identities	Gaps	Strand
34.2 bits(17)	156	20/21(95%)	0/21(0%)	Plus/Minus

```
Query  5     TGCTACGCATATATGCAGAAA  25
             |||||||||||||| ||||||
Sbjct  2554  TGCTACGCATATATACAGAAA  2534
```

A. incomplexa

GenBank Graphics Distance tree of results MSA Viewer

Scientific Name	Max Score	Total Score	Query Cover	E value	Per. Ident	Acc. Len	Accession
Alternaria inco…	50.1	50.1	100%	0.003	100.00%	516	XM_051436542.1
Manduca sexta	40.1	40.1	80%	2.5	100.00%	2509	XM_030173978.2
Campylobacter …	36.2	36.2	72%	40	100.00%	1520669	CP031611.1
Campylobacter …	36.2	36.2	72%	40	100.00%	1520165	CP065357.1
Campylobacter …	36.2	36.2	72%	40	100.00%	1509100	CP063536.1
Topomyia yanb…	36.2	36.2	72%	40	100.00%	8619	XM_058977247.1

Download GenBank Graphics

Alternaria incomplexa uncharacterized protein (J4E90_007000), partial mRNA

Sequence ID: XM_051436542.1 Length: 516 Number of Matches: 1

Range 1: 20 to 44 GenBank Graphics ▼ Next Match ▲ Previous Match

Score	Expect	Identities	Gaps	Strand
50.1 bits(25)	0.003	25/25(100%)	0/25(0%)	Plus/Minus

```
Query  1   TTGCGGGACATGCTAAGGTCCGAAA  25
           |||||||||||||||||||||||||
Sbjct  44  TTGCGGGACATGCTAAGGTCCGAAA  20
```

Download GenBank Graphics

PREDICTED: Manduca sexta uncharacterized LOC115447060 (LOC115447060), mRNA

Sequence ID: XM_030173978.2 Length: 2509 Number of Matches: 1

Range 1: 476 to 495 GenBank Graphics ▼ Next Match ▲ Previous Match

Score	Expect	Identities	Gaps	Strand
40.1 bits(20)	2.5	20/20(100%)	0/20(0%)	Plus/Minus

```
Query  6    GGACATGCTAAGGTCCGAAA  25
            ||||||||||||||||||||
Sbjct  495  GGACATGCTAAGGTCCGAAA  476
```

A. metachromatica

GenBank Graphics Distance tree of results MSA Viewer

Scientific Name	Max Score	Total Score	Query Cover	E value	Per. Ident	Acc. Len	Accession
Alternaria meta…	50.1	50.1	100%	0.003	100.00%	2148	XM_049328263.1
Chitinophaga s…	42.1	42.1	84%	0.64	100.00%	8674373	CP128362.1
Alligator missis…	38.2	38.2	76%	10	100.00%	625	XM_059718219.1
uncultured Allor…	36.2	36.2	72%	40	100.00%	4533630	OY288189.1
uncultured mari…	36.2	36.2	72%	40	100.00%	187348	LC629466.1
Heterocephalus…	36.2	36.2	72%	40	100.00%	45364800	OZ001136.1

Download GenBank Graphics

Alternaria metachromatica uncharacterized protein (J4E83_001306), partial mRNA

Sequence ID: XM_049328263.1 Length: 2148 Number of Matches: 1

Range 1: 501 to 525 GenBank Graphics ▼ Next Match ▲ Previous Match

Score	Expect	Identities	Gaps	Strand
50.1 bits(25)	0.003	25/25(100%)	0/25(0%)	Plus/Minus

```
Query  1    ATCATGACTGCCAGAGGTTCGCAAA  25
            |||||||||||||||||||||||||
Sbjct  525  ATCATGACTGCCAGAGGTTCGCAAA  501
```

Download GenBank Graphics

Chitinophaga sp. LS1 chromosome, complete genome

Sequence ID: CP128362.1 Length: 8674373 Number of Matches: 1

Range 1: 4895499 to 4895519 GenBank Graphics ▼ Next Match ▲ Previous Match

Score	Expect	Identities	Gaps	Strand
42.1 bits(21)	0.64	21/21(100%)	0/21(0%)	Plus/Plus

```
Query  5        TGACTGCCAGAGGTTCGCAAA  25
                |||||||||||||||||||||
Sbjct  4895499  TGACTGCCAGAGGTTCGCAAA  4895519
```

A. novae-zelandiae

GenBank Graphics Distance tree of results MSA Viewer

Scientific Name	Max Score	Total Score	Query Cover	E value	Per. Ident	Acc. Len	Accession
Alternaria nova…	50.1	50.1	100%	0.003	100.00%	4077	XM_049403551.1
Daphnia magna	40.1	40.1	80%	2.5	100.00%	2550	XM_032930981.2
Tyto alba	38.2	38.2	76%	10	100.00%	11978	XM_042804486.1
Tyto alba	38.2	38.2	76%	10	100.00%	11571	XM_032992316.2
Tyto alba	38.2	38.2	76%	10	100.00%	12386	XM_042804485.1
Tyto alba	38.2	38.2	76%	10	100.00%	11694	XM_042804488.1

Download GenBank Graphics

Alternaria novae-zelandiae uncharacterized protein (J4E88_009953), partial mRNA

Sequence ID: XM_049403551.1 Length: 4077 Number of Matches: 1

Range 1: 740 to 764 GenBank Graphics ▼ Next Match ▲ Previous Match

Score	Expect	Identities	Gaps	Strand
50.1 bits(25)	0.003	25/25(100%)	0/25(0%)	Plus/Plus

```
Query  1    GGCAGAATGCAACTTGTATCGGAAA  25
            |||||||||||||||||||||||||
Sbjct  740  GGCAGAATGCAACTTGTATCGGAAA  764
```

Download GenBank Graphics

PREDICTED: Daphnia magna uncharacterized LOC116924451 (LOC116924451), mRNA

Sequence ID: XM_032930981.2 Length: 2550 Number of Matches: 1

Range 1: 731 to 750 GenBank Graphics ▼ Next Match ▲ Previous Match

Score	Expect	Identities	Gaps	Strand
40.1 bits(20)	2.5	20/20(100%)	0/20(0%)	Plus/Plus

```
Query  6    AATGCAACTTGTATCGGAAA  25
            ||||||||||||||||||||
Sbjct  731  AATGCAACTTGTATCGGAAA  750
```

A. panax

GenBank Graphics Distance tree of results MSA Viewer

Scientific Name	Max Score	Total Score	Query Cover	E value	Per. Ident	Acc. Len	Accession
Musa acuminat…	38.2	38.2	76%	10	100.00%	3031	XM_065178779.1
Musa acuminat…	38.2	38.2	76%	10	100.00%	3027	XM_065179093.1
Erwinia sp. QL-Z3	38.2	38.2	76%	10	100.00%	4926645	CP037950.1
Musa acuminat…	38.2	38.2	76%	10	100.00%	39038070	HG996476.1
Erwinia billingia…	38.2	38.2	76%	10	100.00%	5100167	FP236843.1
Inhella inkyong…	38.2	38.2	76%	10	100.00%	4084412	CP040709.1

Download GenBank Graphics

PREDICTED: Musa acuminata AAA Group leucine-rich repeat receptor protein kinase HPCA

Sequence ID: XM_065178779.1 Length: 3031 Number of Matches: 1

See 1 more title(s) See all Identical Proteins(IPG)

Range 1: 2334 to 2352 GenBank Graphics Next Match Previous Match

Score	Expect	Identities	Gaps	Strand
38.2 bits(19)	10	19/19(100%)	0/19(0%)	Plus/Plus

```
Query  2     CGACAGCAATCTCGGCCAT  20
             |||||||||||||||||||
Sbjct  2334  CGACAGCAATCTCGGCCAT  2352
```

Download GenBank Graphics

PREDICTED: Musa acuminata AAA Group leucine-rich repeat receptor protein kinase HPCA

Sequence ID: XM_065179093.1 Length: 3027 Number of Matches: 1

Range 1: 2229 to 2247 GenBank Graphics Next Match Previous Match

Score	Expect	Identities	Gaps	Strand
38.2 bits(19)	10	19/19(100%)	0/19(0%)	Plus/Plus

```
Query  2     CGACAGCAATCTCGGCCAT  20
             |||||||||||||||||||
Sbjct  2229  CGACAGCAATCTCGGCCAT  2247
```

A. porri

GenBank Graphics Distance tree of results MSA Viewer

Scientific Name	Max Score	Total Score	Query Cover	E value	Per. Ident	Acc. Len	Accession
Heterocephalus…	40.1	40.1	80%	2.5	100.00%	97773000	OZ001112.1
Heterocephalus…	40.1	40.1	80%	2.5	100.00%	97502687	OZ001069.1
Malus sylvestris	38.2	38.2	76%	10	100.00%	1395	XM_050297331.1
Trypanosoma g…	38.2	38.2	76%	10	100.00%	2280	XM_009309339.1
Planococcus ko…	38.2	70.4	76%	10	100.00%	3472056	CP013661.2
Topomyia yanb…	38.2	38.2	76%	10	100.00%	1827	XM_058978931.1

Download GenBank Graphics

Heterocephalus glaber genome assembly, chromosome: 4

Sequence ID: OZ001112.1 Length: 97773000 Number of Matches: 1

Range 1: 45768597 to 45768616 GenBank Graphics Next Match Previous Match

Score	Expect	Identities	Gaps	Strand
40.1 bits(20)	2.5	20/20(100%)	0/20(0%)	Plus/Minus

```
Query  6         TAAGGCATGGAAGCAGCAAA  25
                 ||||||||||||||||||||
Sbjct  45768616  TAAGGCATGGAAGCAGCAAA  45768597
```

Download GenBank Graphics

Heterocephalus glaber genome assembly, chromosome: 4

Sequence ID: OZ001069.1 Length: 97502687 Number of Matches: 1

Range 1: 45528861 to 45528880 GenBank Graphics Next Match Previous Match

Score	Expect	Identities	Gaps	Strand
40.1 bits(20)	2.5	20/20(100%)	0/20(0%)	Plus/Minus

```
Query  6         TAAGGCATGGAAGCAGCAAA  25
                 ||||||||||||||||||||
Sbjct  45528880  TAAGGCATGGAAGCAGCAAA  45528861
```

A. postmessia

GenBank Graphics Distance tree of results MSA Viewer

Scientific Name	Max Score	Total Score	Query Cover	E value	Per. Ident	Acc. Len	Accession
Alternaria post…	50.1	50.1	100%	0.003	100.00%	3423	XM_051727408.1
[Pantoea] beijin…	36.2	36.2	72%	40	100.00%	4107390	CP071409.1
Misgurnus angu…	36.2	36.2	72%	40	100.00%	14142	XM_055198209.1
Paramisgurnus …	36.2	36.2	72%	40	100.00%	13818	XM_065269756.1
Volvox carteri f. …	36.2	36.2	72%	40	100.00%	1761	XM_002951411.1
Tigriopus califor…	36.2	36.2	72%	40	100.00%	3689	XM_059223979.1

Download GenBank Graphics

Alternaria postmessia uncharacterized protein (J4E82_001091), partial mRNA

Sequence ID: XM_051727408.1 Length: 3423 Number of Matches: 1

Range 1: 1552 to 1576 GenBank Graphics Next Match Previous Match

Score	Expect	Identities	Gaps	Strand
50.1 bits(25)	0.003	25/25(100%)	0/25(0%)	Plus/Minus

```
Query  1     TCGCGTCCATGTACCTTGAGACAAA  25
             |||||||||||||||||||||||||
Sbjct  1576  TCGCGTCCATGTACCTTGAGACAAA  1552
```

Download GenBank Graphics

[Pantoea] beijingensis strain JZB2120001 chromosome, complete genome

Sequence ID: CP071409.1 Length: 4107390 Number of Matches: 1

Range 1: 3088295 to 3088312 GenBank Graphics Next Match Previous Match

Score	Expect	Identities	Gaps	Strand
36.2 bits(18)	40	18/18(100%)	0/18(0%)	Plus/Plus

```
Query  1        TCGCGTCCATGTACCTTG  18
                ||||||||||||||||||
Sbjct  3088295  TCGCGTCCATGTACCTTG  3088312
```

A. rosae

GenBank Graphics Distance tree of results MSA Viewer

Scientific Name	Max Score	Total Score	Query Cover	E value	Per. Ident	Acc. Len	Accession
Alternaria rosae	50.1	50.1	100%	0.003	100.00%	1353	XM_046175248.1
Truncatella ang…	38.2	38.2	76%	10	100.00%	2802	XM_046108182.1
Burkholderia sp.…	36.2	36.2	72%	40	100.00%	3809753	CP142886.1
Burkholderia or…	36.2	36.2	72%	40	100.00%	3532883	CP000958.1
Burkholderia ce…	36.2	36.2	72%	40	100.00%	3668000	CP019674.1
Burkholderia ce…	36.2	36.2	72%	40	100.00%	6028390	CP019666.1

Download GenBank Graphics

Alternaria rosae uncharacterized protein (BKA58DRAFT_471855), partial mRNA

Sequence ID: XM_046175248.1 Length: 1353 Number of Matches: 1

Range 1: 715 to 739 GenBank Graphics Next Match Previous Match

Score	Expect	Identities	Gaps	Strand
50.1 bits(25)	0.003	25/25(100%)	0/25(0%)	Plus/Minus

```
Query  1    CAACTGCGTCATCGCGGTAATGAAA  25
            |||||||||||||||||||||||||
Sbjct  739  CAACTGCGTCATCGCGGTAATGAAA  715
```

Download GenBank Graphics

Truncatella angustata uncharacterized protein (BKA67DRAFT_663437), partial mRNA

Sequence ID: XM_046108182.1 Length: 2802 Number of Matches: 1

Range 1: 1108 to 1126 GenBank Graphics Next Match Previous Match

Score	Expect	Identities	Gaps	Strand
38.2 bits(19)	10	19/19(100%)	0/19(0%)	Plus/Minus

```
Query  1     CAACTGCGTCATCGCGGTA  19
             |||||||||||||||||||
Sbjct  1126  CAACTGCGTCATCGCGGTA  1108
```

A. ventricose

GenBank Graphics Distance tree of results MSA Viewer

Scientific Name	Max Score	Total Score	Query Cover	E value	Per. Ident	Acc. Len	Accession
Hylaeus anthra…	38.2	38.2	76%	10	100.00%	5334	XM_054143142.1
Hylaeus volcani…	38.2	38.2	76%	10	100.00%	5309	XM_054127243.1
Hylaeus anthra…	38.2	38.2	76%	10	100.00%	5185	XM_054143143.1
Hylaeus volcani…	38.2	38.2	76%	10	100.00%	4667	XM_054127244.1
Hylaeus volcani…	38.2	38.2	76%	10	100.00%	5406	XM_054127242.1
Hylaeus anthra…	38.2	38.2	76%	10	100.00%	5338	XM_054143141.1

Download GenBank Graphics

PREDICTED: Hylaeus anthracinus TOX high mobility group box family member 3-like (LOC1

Sequence ID: XM_054143142.1 Length: 5334 Number of Matches: 1

Range 1: 2865 to 2883 GenBank Graphics Next Match Previous Match

Score	Expect	Identities	Gaps	Strand
38.2 bits(19)	10	19/19(100%)	0/19(0%)	Plus/Plus

```
Query  3     AGTCTTCGGTTGTACGCTA  21
             |||||||||||||||||||
Sbjct  2865  AGTCTTCGGTTGTACGCTA  2883
```

Download GenBank Graphics

PREDICTED: Hylaeus volcanicus TOX high mobility group box family member 3-like (LOC12

Sequence ID: XM_054127243.1 Length: 5309 Number of Matches: 1

Range 1: 3905 to 3923 GenBank Graphics Next Match Previous Match

Score	Expect	Identities	Gaps	Strand
38.2 bits(19)	10	19/19(100%)	0/19(0%)	Plus/Plus

```
Query  3     AGTCTTCGGTTGTACGCTA  21
             |||||||||||||||||||
Sbjct  3905  AGTCTTCGGTTGTACGCTA  3923
```

A. viburni

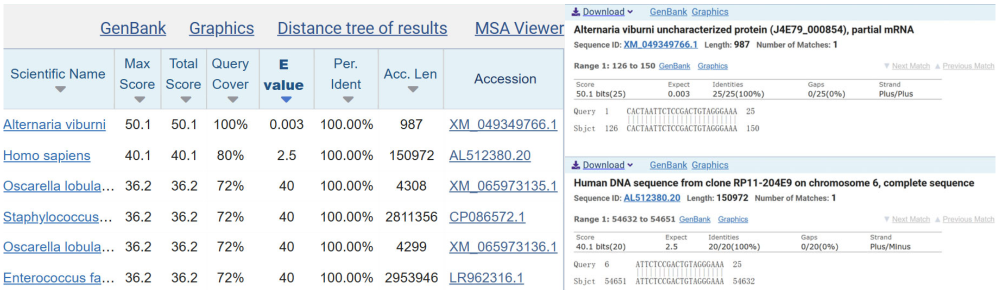

GenBank Graphics Distance tree of results MSA Viewer

Scientific Name	Max Score	Total Score	Query Cover	E value	Per. Ident	Acc. Len	Accession
Alternaria viburni	50.1	50.1	100%	0.003	100.00%	987	XM_049349766.1
Homo sapiens	40.1	40.1	80%	2.5	100.00%	150972	AL512380.20
Oscarella lobula…	36.2	36.2	72%	40	100.00%	4308	XM_065973135.1
Staphylococcus…	36.2	36.2	72%	40	100.00%	2811356	CP086572.1
Oscarella lobula…	36.2	36.2	72%	40	100.00%	4299	XM_065973136.1
Enterococcus fa…	36.2	36.2	72%	40	100.00%	2953946	LR962316.1

Figure S1. Screening Process of Reference-Specific Target Sequences.
